# Mucosa-Associated Bacterial Microbiome of the Gastrointestinal Tract of Weaned Pigs and Dynamics Linked to Dietary Calcium-Phosphorus

**DOI:** 10.1371/journal.pone.0086950

**Published:** 2014-01-23

**Authors:** Evelyne Mann, Stephan Schmitz-Esser, Qendrim Zebeli, Martin Wagner, Mathias Ritzmann, Barbara U. Metzler-Zebeli

**Affiliations:** 1 Institute of Milk Hygiene, Department for Farm Animals and Veterinary Public Health, Vetmeduni Vienna, Vienna, Austria; 2 Institute of Animal Nutrition and Functional Plant Compounds, Department for Farm Animals and Veterinary Public Health, Vetmeduni Vienna, Vienna, Austria; 3 Clinic for Swine, Department for Farm Animals and Veterinary Public Health, Vetmeduni Vienna, Vienna, Austria; 4 Research Cluster ‘Animal Gut Health’, Department for Farm Animals and Veterinary Public Health, Vetmeduni Vienna, Vienna, Austria; 5 Clinic for Swine, Ludwig-Maximilians-Universität München, Munich, Germany; National Institute of Agronomic Research, France

## Abstract

Dietary composition largely influences pig’s gastrointestinal microbiota and represents a useful prophylactic tool against enteric disturbances in young pigs. Despite the importance for host-microbe interactions and bacterial colonization, dietary responses of the mucosa-associated bacterial communities are less well investigated. In the present study, we characterized the mucosa-associated bacterial communities at the *Pars non-glandularis* of the stomach, ileum and colon, and identified shifts in these communities in response to different dietary calcium-phosphorus (Ca-P) contents (100% versus 190% of the Ca and P requirements) in combination with two basal diets (wheat-barley- or corn-based) in weaned pigs. Pyrosequencing of 16S rRNA genes from 93 mucosal samples yielded 447,849 sequences, clustering into 997 operational taxonomic units (OTUs) at 97% similarity level. OTUs were assigned to 198 genera belonging to 14 different phyla. Correlation-based networks revealed strong interactions among OTUs at the various gastrointestinal sites. Our data describe a previously not reported high diversity and species richness at the *Pars non-glandularis* of the stomach in weaned pigs. Moreover, high versus adequate Ca-P content significantly promoted *Lactobacillus* by 14.9% units (1.4 fold change) at the gastric *Pars non-glandularis* (*P* = 0.035). Discriminant analysis revealed dynamic changes in OTU composition in response to dietary cereals and Ca-P contents at all gastrointestinal sites which were less distinguishable at higher taxonomic levels. Overall, this study revealed a distinct mucosa-associated bacterial community at the different gut sites, and a strong effect of high Ca-P diets on the gastric community, thereby markedly expanding our comprehension on mucosa-associated microbiota and their diet-related dynamics in weaned pigs.

## Introduction

Diet is an important environmental factor shaping animal’s microbiota. In pigs, the microbial ecosystem undergoes massive fluctuations in the time after weaning, and pigs are prone to enteric dysbiosis until a stable autochthonous microbiota has been developed [1]. One of the most abundant beneficial bacterial groups in pig’s gastrointestinal tract (GIT) that are reduced after weaning is the genus *Lactobacillus* [2]. A lack of these beneficial bacteria associated with the mucosa allows pathogens to adhere to enterocytes and to proliferate with negative consequences for gut health [3]. In view of the public demand to reduce antibiotic use in animal production, enhancing porcine gut health after weaning by dietary intervention has been receiving more and more attention [4], [5]. Because of their importance for pig’s microbial eubiosis, it was postulated that effective dietary strategies should benefit the lactobacilli community and reduce enterobacterial abundance [1], [6]. Modulation of dietary protein level, carbohydrate composition, and feed additives such as pre- and probiotics are among today’s dietary strategies implemented to support a healthy GIT microbiota in weaned pigs [1], [6].

A promising dietary strategy to modulate pig’s intestinal eubiosis, which received much attention in human research recently using rat models, is dietary calcium and phosphorus (Ca-P). In fact, an improved colonization resistance against intestinal pathogens and promotion of lactobacilli in ileal digesta and at the ileal mucosa has been observed with Ca-P rich diets in rats [7]. In pig nutrition, Ca-P contents, included in the diet at levels above the requirements, are regarded as disadvantageous for weaner pig’s health by potentially compromising gastric barrier function [8]. Yet, evidence emerges that dietary Ca-P may modulate the porcine bacterial microbiota as well [9], [10]. 16S rRNA gene sequencing surveys have deeply enlarged our knowledge on the porcine GIT microbiota and led to a more comprehensive view of the response of the porcine GIT microbiota towards dietary changes [11], [12], [13]; however, studies almost exclusively focused on luminal bacteria so far. Despite the importance of the mucosal community for bacterial intestinal colonization, pathogen resistance, and host-microbiota cross-talk [14], the porcine mucosa-associated microbiota and their diet-related changes at different GIT sites were not investigated at this degree until now.

The major objectives of this study were to characterize the mucosa-associated bacterial communities in the *Pars non-glandularis* of the stomach, ileum and colon of weaned pigs, and to investigate the impact of dietary Ca-P content in combination with two different basal diets (one based on wheat-barley, the other based on corn) on community shifts at different mucosal GIT sites. The reason behind feeding two different basal diets was the differing carbohydrate fractions of the selected cereals which may interfere in Ca-P effects due to different stimulation of bacteria caused by bacterial substrate (carbohydrate) preferences. We obtained more 16S rRNA gene sequences using Roche/454-sequencing for the mucosa-associated microbiota than previously possible to obtain using cloning and Sanger sequencing [15]–[19], implicating a huge complexity of pig’s mucosal microbiota even at this very young age. Diet-induced community shifts showed a considerable stimulating effect of Ca-rich diets on *Lactobacillus*-OTUs at the *Pars nonglandularis* of the stomach.

## Materials and Methods

### Ethics Statement

All procedures involving animal handling and treatments were approved by the institutional ethics committee of the Vetmeduni Vienna and the national authority according to §8ff of Law for Animal Experiments, Tierversuchsgesetz – TVG (GZ 68.205/0222-II/3b/2011).

### Animals, Diets and Sampling

Thirty-two barrows ((Landrace×Large White)×Piétrain) randomly taken from 16 sows and with similar body weight at weaning (day 28 of age) were used in a 2×2 factorial arrangement of dietary treatments in a block-randomized design. Experimental diets (Table S1) were formulated to meet or exceed current recommendations for nutrient requirements for 10–20 kg pigs [20]. The four diets comprised either wheat and barley or corn as basal cereal sources and contained two levels of Ca-P: i) wheat-barley diet with adequate Ca-P content; ii) wheat-barley diet with high Ca-P content; iii) corn diet with adequate Ca-P content; and iv) corn diet with high Ca-P content. The Ca-P content of the adequate Ca-P diets was formulated to contain 100% of the actual Ca and P requirement [20]. The high Ca-P diets were formulated to contain a Ca-P content of about 190% of the actual Ca and P requirements [20]. The experimental diets were fed *ad libitum* for 14 days. Pigs (average body weight 9.5±0.11 kg) were individually housed in stainless steel metabolism cages with free access to demineralized water. The experiment was divided into four runs that were carried out one after another and every run included eight pigs. Two pigs per run received one of the four experimental diets resulting in eight observations per diet over the whole experiment. Siblings did not receive the same experimental diet. Diets were analyzed for dry matter, crude protein, crude fiber, crude ash, neutral detergent fiber, acid detergent fiber, Ca, P [21], xylose and mixed-linked β-glucan (Megazyme International Ireland Ltd, Bray, Ireland).

On day 15 pigs were anesthetized and euthanized. After opening the visceral cavity, esophagus and rectum were clamped to avoid spilling of gastrointestinal digesta and thus contamination of other intestinal parts. Immediately after removing the GIT from the visceral cavity, stomach, ileum and mid-colon (colon was divided into three equal parts) were separated by clamping to avoid mixing of digesta from adjacent segments of the GIT. Subsequently, intestinal segments were disclosed at the mesentery with sterile instruments and digesta was removed. The luminal sites were individually rigorously washed with sterile ice-cold phosphate-buffered saline until the mucosa was completely cleaned from digesta. The mucosa was rinsed with sterile ice-cold phosphate buffered saline several times to remove remains of free-floating bacteria. Mucosa scrapings from the *Pars non-glandularis* (stomach), ileum and mid-colon were collected aseptically by scraping off the mucosa using scalpel blades. Mucosal scrapings were kept on ice until being stored at −20°C. In total, 31 pigs were sampled (one pig fed the wheat-barley diet with high Ca-P content was excluded because it developed meningitis at the start of experiment), resulting in a total of 93 samples from the *Pars non-glandularis* of the stomach, ileum, and colon.

### DNA Extraction, Preparation of 16S rRNA Gene Amplicon Libraries and Pyrosequencing

Genomic DNA was extracted from 250 mg of mucosal scrapings (stomach, ileum, and colon) using the PowerSoil DNA Isolation kit (MoBio Laboratories, Carlsbad, CA, USA) according to manufacturer’s instructions. DNA concentration was determined by a Qubit fluorometer (Invitrogen, Carlsbad, CA, USA) and adjusted to 25 ng/µl. 16S rRNA genes were amplified using FLX 454 one way read fusion primers with the template specific sequence F27–AGAGTTTGATCCTGGCTCAG [22] and R357–CTGCTGCCTYCCGTA [23] targeting the V1–V2 hypervariable region of the 16S rRNA gene (Lib-L kit, Primer A, Primer B, Roche 454 Life Science, Branford, CT, USA) (Table S2). For each sample, a PCR mix of 50 µl was prepared containing 1× Fast Start High Fidelity Buffer, 2.5 U High Fidelity Enzyme, 200 µM dNTPs (Roche Diagnostics, Mannheim, Germany), 0.4 µM barcoded primers (Eurofins MWG, Ebersberg, Germany), 2.5 mM MgCl2, PCR-grade water (Roche Diagnostics) and 125 ng total genomic DNA. Thermal cycling conditions were initial denaturation at 95°C for 3 min followed by 38 cycles of denaturation at 95°C for 45 s, annealing at 56°C for 45 s and extension at 72°C for 1 min with a final extension of 7 min at 72°C. Amplicons were purified and collected using a denaturing HPLC on a WAVE apparatus (Transgenomic Inc., Omaha, NE, USA) and eluted using a linear gradient (typically 12–17%) of acetonitrile in 0.1 M triethylammoniumacetate over 10 min at 50°C. Amplicon DNA was purified on NucleoFast® 96 PCR plates (Macherey-Nagel, Düren, Germany) by using a vacuum pump according to manufacturer’s instructions and eluted in 30 µl elution buffer (Qiagen, Hilden, Germany). Amplicon DNA concentrations were determined using the Quant-iT™ PicoGreen® dsDNA Assay Kit (Life Technologies, Carlsbad, CA, USA) according to manufacturer’s instructions. After quantification, 30 barcode labeled amplicons were pooled equimolar and analyzed on a 2100 Bio Analyzer (Agilent Technologies, Waldbronn, Germany) using a DNA 7500 kit (Agilent Technologies). Sequencing of the equimolar pool of 30 samples on a quarter Pico Titer Plate was performed using the GS FLX Titanium Sequencing Kit XLR70 (Roche 454 Life Science) according to manufacturer’s instructions. Library preparation and sequencing was performed at the Center for Medical Research, Core Facility Molecular Biology, Medical University of Graz (Graz, Austria).

### Processing, Phylogenetic Assignment of Sequence Reads and Statistics

All reads derived from pyrosequencing (93 samples; 447,849 reads) were processed together using the software package mothur [24], according to the procedure described by Schloss et al. [25]. Primers, barcode sequences and sequences of low quality and length were trimmed with a minimum average quality score of 35 (using a window size of 50 bp) and a minimum length of reads of 162 bp (Text S1). Sequencing error was reduced using the “pre.cluster” algorithm. Chimeric sequences were excluded using “chimera.uchime”. 408,171 sequences (91%) with a median length of 198 bp passed the quality control. The remaining high quality reads were classified using the RDP naïve Bayesian rRNA classifier (confidence threshold = 80%) [26] with the SILVA reference database [27]. Based on this alignment, uncorrected pairwise distances were calculated using “dist.seqs”. These pairwise distances served as input for the assignment to operational taxonomic units (OTUs). OTUs were assigned using a cutoff ( = distance limit) of 0.03. OTUs with less than 10 sequences assigned were removed (4,658 OTUs were removed; the mean sequence number was 2.2). Consensus taxonomy for OTUs was given with the “classify.otu” command and the OTUs were split up per sample. Before estimating the total number of species in GIT sites, data were normalized by a random selection of the same sequence number per sample. The sequence number was determined based upon the sample with the least number of sequences (*n* = 1,291 sequences). The “summary.single” command was used for calculating the nonparametric estimate Chao1, the diversity indices Shannon and Simpson and Bray-Curtis similarity. Raw pyrosequencing data are available in the EMBL SRA database under the accession number ERP002312.

For generating summarized, smooth rarefaction curves per GIT site, an algorithm previously described [28] was modified. A table was generated including the number of OTUs identified for every 20 sequences up to 500 followed by every 500 after that. At every point where a sample ’dropped out’, calculated weighted values were multiplied to balance the overall mean values. The weighted value was equal to the overall mean at the position of the drop-out, divided by the mean at the position before.

The assigned OTUs and the diversity indices were subjected to ANOVA using the PROC MIXED of SAS (Text S1) (Statistical Analysis System 9.2, SAS Inst. Inc., Cary, NC, USA). The procedure PROC CORR was used to estimate Pearson’s correlation coefficient between gastric, ileal and colonic pH and OTU abundance at the mucosa of the *Pars non-glandularis* in the stomach, ileum and colon. Significance was declared at *P≤*0.05 and 0.05<*P≤*0.10 was defined as a trend. Results are presented as least squares means ± standard error of the mean.

Each OTU containing >1,000 sequences was blasted against the NCBI GenBank excluding uncultured/environmental sample sequences in the search set. In total 50 OTUs were blasted and annotated with its closest reference strain, accession number and sequence similarity. Correlation networks were created in MENAP (http://ieg2.ou.edu/MENA; molecular ecological network analysis pipeline) [29] and visualized with Cytoscape version 3.0 [30]. Heatmaps were created using JColorGrid [31]. For the illustration of microbial shifts on community level discriminant analysis was done in JMP Pro (SAS Institute, NC, USA) with the 50 most abundant OTUs as covariates and diet as the categorical variable.

## Results

### Pyrosequencing Data, OTU Classification and Phylum Affiliation

All reads deriving from pyrosequencing of 93 samples were processed together. In total, 408,171 sequences (91%) with a median length of 198 bp passed the quality control. On average, 4,437 sequences per sample were obtained. Throughout all samples, 997 OTUs could be assigned with >10 sequences per OTU (4,658 OTUs with <10 sequences were excluded). This OTU classification was used for all further downstream analyses. 50 OTUs contained >1,000 sequences (up to 65,633 sequences). In Table 1, the 50 most abundant OTUs are listed including sequence number, relative abundance and closest reference strain and similarity (NCBI BLAST). All closest reference strains among cultivated bacteria of OTUs were previously described to belong to the commensal porcine GIT microbiota [17].

**Table 1:** The 50

OTU	No.ofsequences	Relativeabundance	Closest reference strain (GenBank accession no.)^1^	Similarity
351	65633	16.5%	*Helicobacter rappini* (AY034817)	99%
1	47856	12.0%	*Lactobacillus johnsonii* (JN012223)	100%
2	19108	4.8%	*Lactobacillus amylovorus* (EF120375)	100%
12	18037	4.5%	*Prevotella copri* (AB649279)	100%
4	16732	4.2%	*Lactobacillus delbrueckii* (AB680073)	100%
5	10444	2.6%	*Prevotella copri* (AB649279)	97%
8	9484	2.4%	*Prevotella copri* (AB649279)	96%
20	7407	1.9%	*Escherichia coli* (FN821375)	99%
3	7266	1.8%	*Lactobacillus mucosae* (AB425938)	100%
41	7086	1.8%	*Clostridium mayombei* (FR733682)	99%
23	6890	1.7%	*Pseudomonas trivialis* (HF585483)	100%
624	6849	1.7%	*Campylobacter lanienae* (JX912520)	99%
3685	6633	1.7%	*Helicobacter suis* (AB498800)	98%
26	6282	1.6%	*Prevotella stercore*a (NR_041364)	98%
22	6233	1.6%	*Bacteroides dorei* (AB714352)	99%
2498	5091	1.3%	*Prevotella ruminicula* (AF218618)	99%
16	4946	1.2%	*Prevotella copri* (AB649279)	95%
11	4792	1.2%	*Streptococcus alactolyticus* (EU728776)	99%
383	4119	1.0%	*Campylobacter jejuni* (JX912519)	99%
21	3637	0.9%	*Bacteroides massiliensis* (AB510703)	99%
6	3583	0.9%	*Lactobacillus reuteri* (GU292563)	100%
1439	3366	0.8%	*Acinetobacter johnsonii* (EU337121)	100%
1853	3094	0.8%	*Clostridium rectum* (X77850)	99%
4808	2816	0.7%	*Acinetobacter johnsonii* (EU123856)	99%
18	2491	0.6%	*Prevotella copri* (NR_040877)	95%
34	2370	0.6%	*Clostridium perfringens* (JX267106)	99%
2489	2196	0.6%	*Prevotella ruminicula* (AF218618)	99%
52	2177	0.5%	*Fusobacterium gonidiaformans* (GU429478)	99%
4692	2132	0.5%	*Acinetobacter johnsonii* (JQ435689)	100%
1151	2105	0.5%	*Citrobacter freundii* (KC211308)	99%
14	2056	0.5%	*Clostridium* sp. 826 (AB739699)	99%
95	2049	0.5%	*Prevotella oulorum* (NR_029147)	90%
35	2044	0.5%	*Haemophilus parainfluenzae* (EU083530)	99%
49	2029	0.5%	*Prevotella stercorea* (NR_041364)	99%
63	1819	0.5%	*Haemophilus* sp. D191-1 (FJ463822)	94%
945	1809	0.5%	*Prevotella ruminicola* (AF218618)	99%
29	1688	0.4%	*Proteus mirabilis* (KC211292)	99%
27	1642	0.4%	*Lachnospiraceae bacterium* DJF_RR61 (EU728764)	98%
56	1517	0.4%	*Acidovorax ebreus* (CP001392)	99%
360	1410	0.4%	*Bacteroides dorei* (NR_041351)	99%
4809	1345	0.3%	*Acinetobacter bouvetii* (KC514127)	99%
155	1240	0.3%	*Prevotellaceae bacterium* DJF_VR15 (EU728784)	99%
24	1185	0.3%	*Clostridium* sp. 2ER371.1 (JQ248565)	99%
51	1151	0.3%	*Streptococcus porcorum* (FN908166)	100%
137	1138	0.3%	*Faecalibacterium prausnitzii* (HQ457032)	99%
89	1137	0.3%	*Eubacterium* sp. F1 (EU281854)	86%
36	1121	0.3%	*Clostridiales bacterium* 80/4 (FJ748580)	99%
59	1121	0.3%	*Staphylococcus epidermidis* (KC443110)	100%
7	1117	0.3%	*Lactobacillus mucosae* (EU728797)	99%
30	1068	0.3%	*Xylanibacter oryzae* (AB588018)	87%

All OTUs were blasted against NCBI GenBank nr. Closest reference strains, accession numbers and similarity values are listed.

1 BlastN against the NCBI nr excluding uncultured/environmental sample sequences.

**Figure 1:**
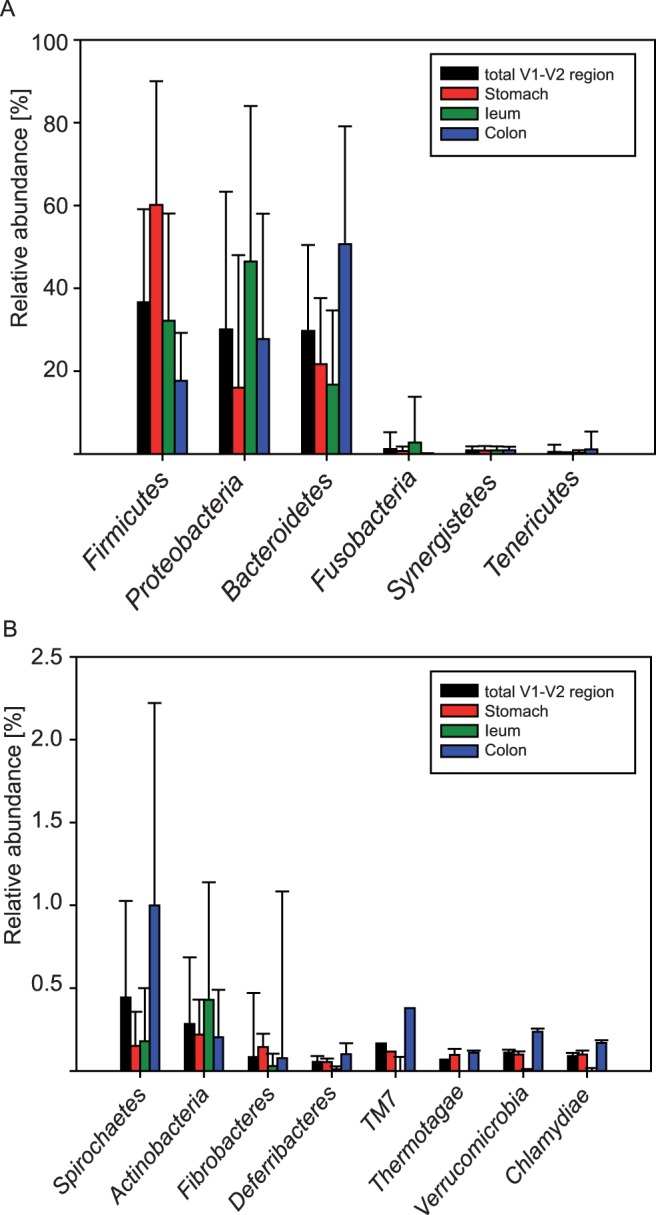
Relative abundances of bacterial phyla attached to the gastrointestinal mucosa independent of diet. (A) Phyla detected in stomach, ileum and colon mucosa samples with >0.5% mean abundance were shown for the total V1–V2 region and for each gastrointestinal site separately. (B) Relative abundances of rare phyla. Error bars represent standard deviation from the mean.

Throughout all GIT sites 14 phyla were identified, *Firmicutes, Proteobacteria* and *Bacteroidetes* being the most abundant ones: 96% of all reads affiliated to these three phyla. In the *Pars non-glandularis* of the stomach *Firmicutes*, in the ileum *Proteobacteria*, and in the colon *Bacteroidetes* was the dominating mucosa-associated phylum (Figure 1).

### Assessment of Diversity and Surveys of the Microbial Community Structure in the Porcine GIT

**Figure 2:**
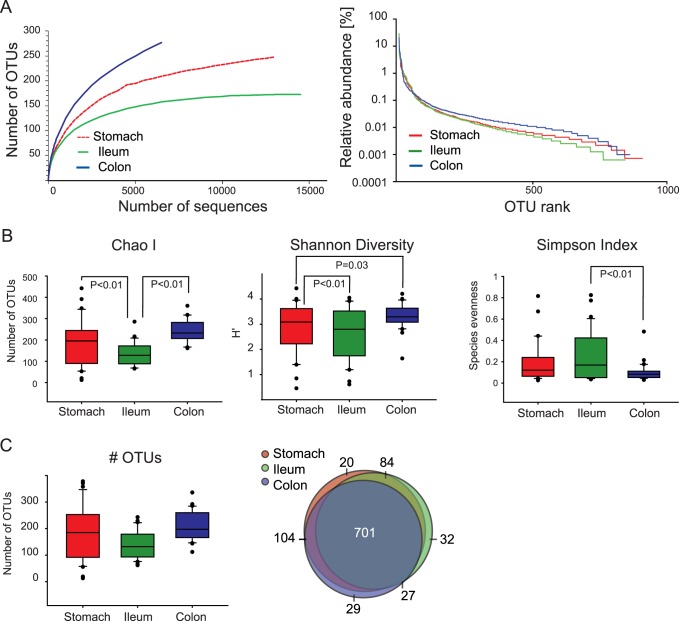
Diversity of mucosa-associated bacteria. (A) Rarefaction and rank abundance curves based on an OTU definition threshold of 0.03 16S rRNA distance are shown. Rarefaction and rank abundance curves were calculated for each sample and depicted as mean per gastrointestinal site (*Pars non-glandularis* of the stomach, ileum and colon mucosa). Sequence numbers of samples were normalized by random selection before calculation. (B) Species richness and diversity estimates for bacteria at gastrointestinal mucosa. Significant differences between GIT sites, calculated with PROC MIXED of SAS, were listed. Significance was declared at *P≤*0.05. (C) Number of OTUs detected per gastrointestinal site and Venn diagram showing the number of shared OTUs between GIT sites. The size of the circles is in proportion to the number of OTUs detected in each gastrointestinal site.

Before performing diversity and richness estimations of GIT communities, the reads of all samples were normalized to the lowest number of sequences in a sample (n = 1,291 sequences) by random selection. Diversity estimations for the *Pars non-glandularis* of stomach, ileum and colon are depicted in Figure 2A: Rank abundance curves indicated that samples contained a low proportion of highly abundant OTUs, whereas the bulk of the present diversity was composed of rare organisms. 29 OTUs in the *Pars non-glandularis* of the stomach, 30 OTUs in ileum and 18 OTUs in colon reached relative abundances of >0.5%. For the determination of completeness of the diversity sequenced, rarefaction curves were calculated for all samples and summarized to GIT sites means. They revealed high diversity coverage, particularly for stomach and ileum samples, with rarefaction curves reaching asymptotes. The trend in rarefaction curves towards increased species richness in the colon and the higher richness at the *Pars non-glandularis* of the stomach vs. ileum were supported by Chao1 (*P*<0.01, PROC MIXED of SAS; Figure 2B). The Simpson index differed between colon vs. ileum (*P*<0.01, PROC MIXED of SAS), but not for stomach vs. ileum and stomach vs. colon. The Shannon index, reflecting both richness and evenness, differed between colon vs. ileum and colon vs. stomach, but not for stomach vs. ileum (*P*<0.01 for colon vs. ileum, *P* = 0.03 for colon vs. stomach, PROC MIXED of SAS). Diversity indices were not different among basal cereal diets and Ca-P contents for all GIT sites (*P*>0.1, PROC MIXED of SAS). Figure 2C depicts the actual number of OTUs detected per GIT site after quality control. Interestingly, at the *Pars non-glandularis* of the stomach ∼185 OTUs were found; however, gastric diversity had a high variance (13–378 OTUs) and was not influenced by diet (*P*>0.1, PROC MIXED of SAS). In the ileum and colon, 132 and 198 OTUs were assigned, respectively. The Venn diagram of Figure 2C displays a low number of unique GIT-site OTUs and a high overlap-pattern for all GIT sites. From 997 OTUs assigned throughout all samples, 701 OTUs (70%) were shared between the mucosa of the *Pars non-glandularis* of the stomach, ileum and colon. Despite the high number of overlapping OTUs, the Bray-Curtis similarity showed clear differences between GIT sites, indicating individual microbial community structures (Figure S1).

**Figure 3:**
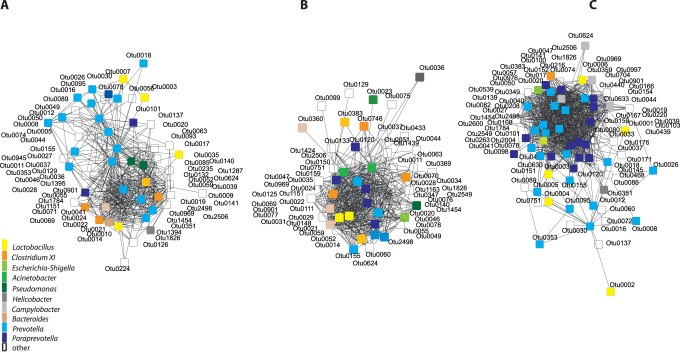
Correlation networks for the microbial communities at the mucosa of (A) the *Pars non-glandularis* of the stomach, (B) the ileum and (C) the colon. The network depicts correlations between the top 10% of all OTUs per GIT site (r >0.5, *P*<0.001). OTUs belonging to the 10 most abundant genera are shown in the same color. Correlation networks were calculated in MENAP (http://ieg2.ou.edu/MENA; molecular ecological network analysis pipeline) and visualized with Cytoscape.

Gastric pH-values (Figure S2) weakly correlated with OTUs detected at the *Pars non-glandularis* (r = 0.39, *P* = 0.03, PROC CORR of SAS). To reveal interactions among OTUs, a correlation-based network was built for each GIT site (r>0.5, *P*<0.001, Figure 3). Network analysis were performed with reduced datasets (top 10% of all OTUs per GIT site) to improve pattern visualization. Additionally, pairwise correlation was calculated and depicted for the 20 most abundant OTUs at each GIT site (Figure S3). Network topology revealed correlations at all GIT sites, with a remarkably high correlation pattern of *Prevotella/Paraprevotella*-OTUs in the *Pars non-glandularis* of the stomach. A very high correlation with r>0.9 was calculated for correlation between *Bacteroides*-OTU 21 and *Bacteroides*-OTU 22, between *Acinetobacter*-OTU 4808 and *Acinetobacter*-OTU 1439, as well as between *Escherichia*-OTU 20 and *Bacteroides-*OTU 22 in the stomach. Interestingly, in the *Pars non-glandularis* lactobacilli correlated only weakly in the stomach, indicating a varying species-abundance pattern in the colonization of *Lactobacillus* due to inter-animal differences in the lactobacilli community. The weak correlation among *Lactobacillus*-OTUs also indicates that different *Lactobacillus* species can occupy the same ecological niche in individual pigs. The same two *Bacteroides*-OTUs (i.e., OTU 21 and OTU 22) that highly correlated in the stomach, also highly correlated (r>0.9) in the ileum. In the colon, the highest denseness of network correlations could be described, with high correlation among *Prevotella/Paraprevotella*. Using pairwise correlation analysis, the highest interaction was shown between *Prevotella*-OTU 12 and *Prevotella*-OTU 16 and between *Lactobacillus*-OTU 4 and *Lachnospira*-OTU 27 in the colon (r>0.6).

Due to the importance of *Lactobacillus* for gut health in weaned pigs and the weak correlation of *Lactobacillus*-OTUs, a maximum likelihood tree including OTUs and closest reference full-length sequences was built for an approximate phylogenetic placement of *Lactobacillus* OTUs (Figure S4). The locations of the OTUs in the tree confirmed highly diverse *Lactobacillus* species in our samples, clustering to 22 different reference full-length sequences.

### GIT Site-related Shifts on OTU and Genus Level

**Figure 4:**
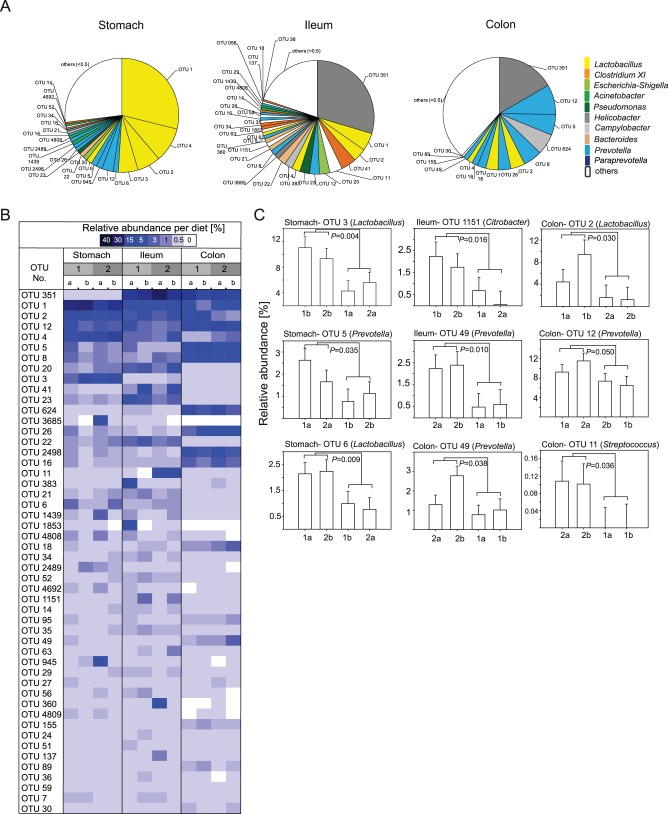
Relative abundance of OTUs per GIT site and diet-related shifts. (A) OTUs with an abundance of >0.5% are shown per GIT site. OTUs with <0.5% abundance were summed up and denoted as “others (<0.5%)”. (B) Heatmap with relative abundances of the 50 most abundant OTUs in the gastrointestinal tract of pigs fed wheat-barley or corn diets including adequate or high Ca-P. Diets were abbreviated as follows: 1a) Wheat-barley diet with adequate Ca-P content; 1b) Wheat-barley diet with high Ca-P content; 2a) corn diet with adequate Ca-P content; 2b) Corn diet with high Ca-P content. (C) Diet-induced shifts on OTU level that reached statistical significance were shown. Diets were abbreviated as follows: 1a) wheat-barley diet with adequate Ca-P content; 1b) wheat-barley diet with high Ca-P content; 2a) corn diet with adequate Ca-P content; and 2b) corn diet with high Ca-P content. Significant differences between GIT sites are calculated with PROC MIXED of SAS. Significance was declared at *P≤*0.05.

Relative OTU abundances per GIT site are shown in Figure 4A. In total, the 50 most abundant OTUs accounted for 72.9% of all sequences and 64% of the 50 most abundant OTUs differed significantly in their relative abundances over all GIT sites (details in Table S3). At the *Pars non-glandularis* of the stomach four highly abundant OTUs (relative abundance >5%) were present, all matching to *Lactobacillus* reference strains. They accounted for over 50% of sequences at this GIT site and were significantly increased at the *Pars non-glandularis* of the stomach compared to the lower GIT sites. In the stomach, a notable high number of 21 OTUs reached a relative abundance between 5% and 0.5%. The *Escherichia*-OTU was clearly associated with the stomach and ileum, showing a significant decrease in the colon. Interestingly, in the ileum, OTU 351, matching closest to *Helicobacter rappini*, was highly abundant (29.6%). OTU 1 (best BLAST hit: *Lactobacillus johnsonii*) had a relative abundance of 5.1%, followed by 30 OTUs between 5% and 0.5%. In the colon, again OTU 351 was the most abundant (16.6%), followed by four OTUs between 5.4% and 8.6%. These OTUs matched to *Prevotella* and *Campylobacter* species. Although most *Prevotella*-OTUs were more abundant in the colon compared to the other sites, it should be mentioned that some *Prevotella*-OTUs were moderately abundant in the ileum and also affiliated to the *Pars non-glandularis* of the stomach (e.g. OTU 945, 2489).

**Figure 5:**
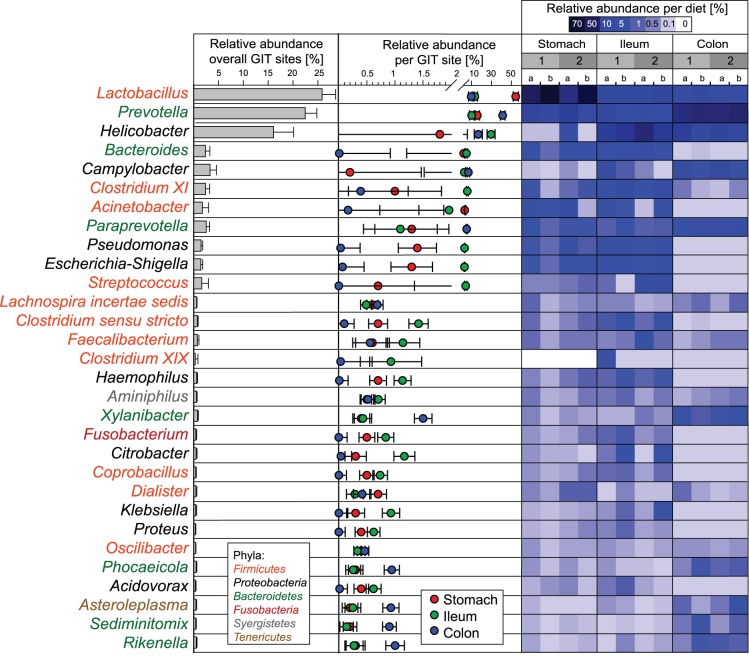
Relative abundances of microbial genera attached to the GIT mucosa. Relative abundances of the 30(*Pars non-glandularis* in the stomach, ileum and colon) and per diet at different GIT sites. Diets were abbreviated as follows: 1) wheat-barley diet; 2) corn diet; a) adequate Ca-P content; and b) high Ca-P content. Genera analyses were based on OTU classification. Significant differences between GIT sites are calculated with PROC MIXED of SAS. Significance was declared at *P≤*0.05. Error bars represent standard deviation from the mean.

Additionally to the overview on phylum and OTU level we combined all OTUs to their respective genus to get insights into cumulated shifts on genus level. Correlation networks and scatterplot matrices of the pairwise correlation analyses (Figure 3, Figure S3) confirmed that highly abundant OTUs belonging to a genus did not correlate in most cases. Several OTUs seemed to be exchangeable within high abundance genera and therefore analyses on genus level are ecologically worthwhile. In total, 997 OTUs could be assigned to 198 genera. In Figure 5, the most abundant genera at the different GIT sites are depicted (details in Table S4).

### Diet-related Shifts on OTU and Genus Level

**Figure 6:**
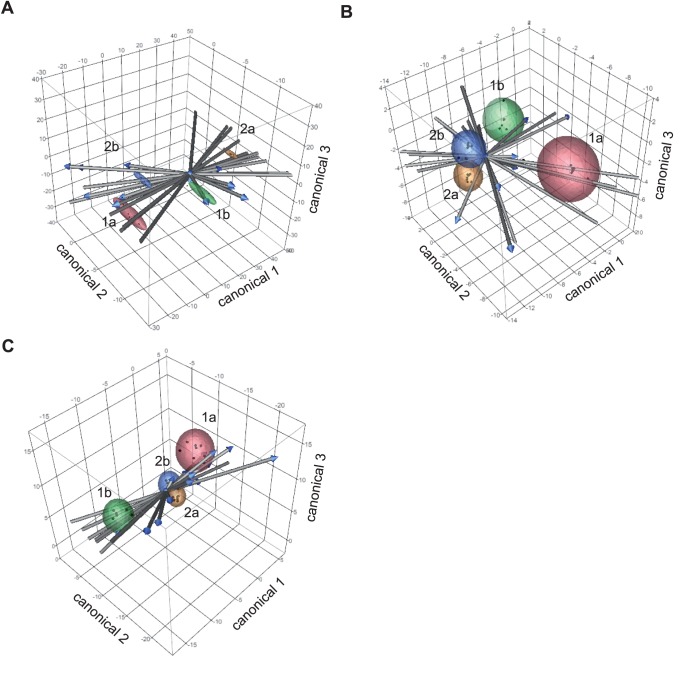
Discriminant analyses with the first 3 principal components for (A) the *Pars non-glandularis* of stomach, (B) ileum and (C) colon, dependent on various diets. For the calculation the 50: 1a) wheat-barley diet with adequate Ca-P content; 1b) wheat-barley diet with high Ca-P content; 2a) corn diet with adequate Ca-P content; and 2b) corn diet with high Ca-P content. For the ileal and colonic mucosa, clusters of corn diets overlapped, indicating less discrimination than in wheat-barley diets.

Using discriminant analysis with the first 3 principal components, community-shifts between the four diets could be clearly depicted (Figure 6). It revealed structural changes in all GIT site-microbiota in response to cereal source and Ca-P level of diets. In ileum and colon, clusters of corn diets with adequate- and high-Ca-P overlapped, indicating less discrimination than clusters of wheat-barley diets.

OTU and genera abundances separated per diet at the three GIT sites are shown in the heatmaps of Figure 4B (detailed abundance coefficients are in Table S5) and Figure 5 (exact values, SEM and *P*-values are available in Table S6), respectively. It is interesting to note that dietary Ca-P content and the composition of the basal diets, i.e. cereal composition, affected different OTUs at the different GIT sites. Significant shifts are seperately shown in Figure 4C: Of particular importance for gut health may be the increase in gastric lactobacilli-OTUs which are typically associated with the porcine gut by high versus adequate Ca-P diets. Together, these lactobacilli-OTUs accounted for 61.6% and 43.1% of the 50 most abundant OTUs at the gastric mucosa with the high Ca-P diets compared to the adquate CaP-diets, respectively. By contrast, there was a general reduction in certain *Prevotella*-OTUs associated with the gastric mucosa by high versus adquate Ca-P diets decreasing the percentage of these OTUs by 7.8% units with high Ca-P diets compared to adequate Ca-P level. More specifically, in the stomach OTU 3 (*Lactobacillus mucosae*) significantly increased by 6.2%-units (2.8 fold change) and OTU 4 (*L. delbrueckii*) tended to be more abundant by 6%-units with high Ca-P diets compared to the adequate Ca-P diets. OTU 5 (*Prevotella copri*) decreased by 1.2% units (2.6 fold change) with high Ca-P diets compared to the adequate Ca-P diets. Dietary Ca-P content also modified the bacterial community at the ileal mucosa but changes in the bacterial abundance were smaller than in the stomach. In the ileum, OTU 1151 (*Citrobacter freundii*) significantly increased by 1.6% units (4.9 fold change) with high Ca-P diets. In the colon, OTU 12 (*Prevotella copri*) showed a significant increase by 3.6% units (1.5 fold change) with high Ca-P vs. adequate Ca-P diets. Shifts on genus level were in accordance with shifts based on OTU level. Particularly, dietary Ca-P content caused measurable effects on the gastric mucosa-associated bacterial genera. At genus level, the stimulating effect of the high Ca-P content on gastric *Lactobacillus* was confirmed increasing this genus by 14.9% units (1.4 fold change) compared to the adequate Ca-P content. In contrast, high vs. adequate Ca-P content decreased gastric *Prevotella* (*P*<0.1), *Campylobacter* (*P*<0.1) and *Phocaeicola* (*P* = 0.042). In the ileum high vs. adequate Ca-P content increased *Clostridium* cluster XI by 6% units (3.3 fold change, *P* = 0.042) and *Citrobacter* by 1.6% units (4.9 fold change, *P* = 0.016), as well as *Clostridium* sensu stricto and *Klebsiella* by 0.8 and 1% units (*P*<0.1). In the colon, high vs. adequate Ca-P content decreased *Campylobacter* by 5.8% units and increased *Lachnospiraceae* incertae sedis by 0.35% units (*P*<0.1).

In the stomach, the basal diet did not cause significant shifts in the microbial community, but OTU 6 (*Lactobacillus reuteri*) showed significant two-way-interaction between Ca-P content and basal diet by a shift of 1.3% units (2.4 fold change). In the ileum, OTU 49 (*Prevotella stercorea*) significantly increased with corn vs. wheat-barley diets (0.2% units, 4 fold change). In the colon, OTU 49 showed a similar response towards the basal diets as in the ileum. OTU 2 (*Lactobacillus amylovorus*) significantly increased by 5.5% units (4.9 fold change), whereas OTU 11 (*Streptococcus alactolyticus*) decreased by 0.1% units with wheat-barley vs. corn diets. Interestingly, basal diet-related shifts on genus level slightly differed from basal diet-related shifts based on OTU level. Whereas the dietary cereal source did not influence the bacterial community in the stomach and ileum, the genus *Streptococcus* increased (*P* = 0.048) by 0.1% units in the colon of pigs fed corn diets compared to pigs fed wheat-barley diets.

## Discussion

The present pyrosequencing study revealed characteristic changes in the mucosal bacterial composition in relation to gut site and dietary treatment, particularly in response to differences in dietary Ca-P level. Interestingly, dietary cereal source only slightly influenced the bacterial mucosal abundance and the effect was restricted to the ileal and colonic mucosa.

Correlation network topology revealed correlations between OTUs at all GIT sites, with the denseness of the correlation network increasing from the stomach to the colon. In particular, correlations for the colonic mucosa-associated bacteria confirm substantial interactions between different bacterial species in the lower part of the GIT. Although we use the term mucosa-associated bacteria, this bacterial community does not directly adhere to the epithelium but colonizes the outer loosely adherent mucus layer, whereas the inner-mucus layer is devoid of bacteria separating bacteria-rich lumen and epithelium [14].

One major finding of the present study was the high diversity of the bacterial community at the *Pars non-glandularis* of the stomach which has not been reported before. We can largely exclude a contamination of the *Pars non-glandularis* with bacteria originating from GIT digesta, because GIT sites were separated by clamping before opening of the gut segments, and the mucosa was rigorously washed and rinsed before sampling. Furthermore, distinct clustering of samples per respective GIT site in the Bray-Curtis similarity matrix provided strong evidence for distinct microbial community structures at the various GIT sites. Due to the particular impact of the mucosa-associated microbiota on the host animal [14], it may be assumed that a highly diverse mucosa-associated microbiota in the *Pars non-glandularis* may be an indicator for gastric health in weaned pigs. Previous studies indicated that bacteria usually found in the lower sites of the GIT are present in digesta of the stomach [10], [32]. Yet, acidic conditions in the stomach usually impair the development of a very diverse community restricting bacterial colonization to acid-tolerant species like *Lactobacillus* sp. [33], [34]. Due to the compartmentalization of pig’s stomach into four distinct mucosal regions, luminal conditions are less acidic at the mucosa of the *Pars non-glandularis,* which is an extension of the esophagus and thus different to conditions in the secretory fundus region of the stomach [35]. It is generally accepted that *Lactobacillus* is the dominating genus in the stomach of pigs. The present range of *Lactobacillus*-OTUs, which dominated at the mucosa of the *Pars non-glandularis* in the current study (i.e., *L. johnsoni, L. amylovorus, L. delbrueckii* and *L. mucosae*) are typical phylotypes inhabiting the porcine GIT [17], [19], [36]. Interestingly. lactobacilli communities correlated only weakly among pigs indicating substantial inter-animal differences and occupation of the same ecological niche by various *Lactobacillus* species in different pigs.

*Prevotellaceae* are known to be a dominant complex carbohydrate-degrading bacterial group in the lower GIT [37] with low abundance or absence in the upper GIT. Yet, our present data demonstrated that the mucosa of the gastric *Pars non-glandularis* in weaned pigs may be inhabited by *Prevotella*. Apparently, *Prevotella* species may tolerate luminal conditions at the mucosa of the *Pars non-glandularis* and the presence of our most abundant *Prevotella-*OTU (*P. copri*) indicate that bacterial degradation of dietary hemicelluloses [38] may already begin at the gastric mucosa of young pigs. Similar to humans, *Helicobacter* species may act as gastric pathogens in pigs [39]. Yet, in our study, *Helicobacter* was rare at the gastric mucosa and was predominately assigned to the ileum and colon. The most abundant OTU (best BLAST hit: *H. rappini*) may cause illness in humans [40], but information for pigs is missing.

Another major finding of this study was that the high Ca-P diets had the potential to distinctly increase the abundance of *Lactobacillus-*OTUs at the mucosa of the *Pars nonglandularis* compared to the adequate Ca-P diets. Especially the promotion of the *Lactobacillus*-OTU related to *L. mucosae* (OTU 3) by 5.2% units in high Ca-P diets is of interest because this species is strongly associated with the mucus layer of the intestinal mucosa in pigs [41] and enforcing their adherence by Ca-P may contribute to reduce attachment of opportunistic pathogens by steric hindrance of mucosal binding sites. Divalent ions can influence bacterial attachment to the mucosa [42]. As recently demonstrated in *in vitro* studies using different cell lines, free Ca^++^ ions appear to promote the mucosal adhesion of various probiotic *Lactobacillus* strains [43], [44]. Due to their general ability to suppress the growth of opportunistic pathogens, such as enterotoxigenic *E. coli,* by very effective bacteriocin and organic acid production [45], [46], [47], Ca-P related promotion of *Lactobacillus* may be beneficial in supporting the gastric barrier. The decrease in gastric gram-negatives (*Prevotella, Campylobacter* and *Phocaeicola*) due to the high Ca-P content might have been caused by competition with *Lactobacillus* for mucosal binding sites. Gram-positive and gram-negative bacteria differ in their cell wall structure; therefore, it might be also feasible that free Ca^++^ ions lowered the binding of gram-negative *Prevotella* species to the mucosa.

Ninety percent of the Ca and P absorption takes place in the small intestine [48], [49]. Nevertheless, more Ca and P was available in the intestinal lumen along the GIT with the high versus adequate Ca-P diets as indicated by the higher faecal excretion of Ca and P in these pigs (data not shown). However, except for the stomach, Ca-P mostly exists in the gut in a dynamic equilibrium of free ions and non-dissociated amorphous Ca-phosphate complex at pH values of above six. Amorphous Ca-phosphate exerts buffering properties and cytoprotective effects in the intestinal lumen by precipitating components in the intestinal lumen, like bile acids, lactic acid and volatile fatty acids [50]. Therefore, acid-sensitive bacterial genera, e.g. *Citrobacter*, might have thriven at the ileal mucosa when more toxic components were precipitated. Because Ca and P are also essential nutrients for bacteria since they are needed for a variety of metabolic processes in the bacterial cell [51], differences in the intestinal availability may have provided growth-advantages for certain bacterial species and genera, such as *Citrobacter, Klebsiella, Clostridium* cluster XI, *Clostridium* sensu strictu at the ileal mucosa. These genera are well known commensals of pig’s ileum and large intestine [12], [17]. In this study, an increase in the ileal mucosa-associated fraction could be achieved with high Ca-P levels. Growth-advantages for Citrobacter and Klebsiella may be ecologically worthwhile. Competition among enterobacteria may reduce pathogenic E.coli which are mainly the causative agent in postweaning diarrhea [17]. Interestingly, commensal Clostridium species belonging to cluster XI have been recently described to be significantly increased in pathologically altered ileocaecal lymph nodes of pigs compared to healthy ones [52]. Higher mucosal abundance of these Clostridium groups might lead to an increased bacterial translocation from the intestinal mucosa to lymph nodes under certain conditions thereby causing an inflammatory immune response in lymphatic tissues.

Dietary carbohydrate composition only sporadically interacted with the dietary Ca-P content indicating that Ca-P effects can be mostly seen independently of the basal diet provided to the pigs. Differences in carbohydrate composition (e.g. starch, arabinoxylan and β-glucan) of the basal diets likely influenced the abundance of *Lactobacillus, Streptococcus* and *Prevotella* OTUs attached to the ileal and/or colonic mucosa. Substrate availability due to progressing digestion, absorption and fermentation, transit time as well as physiological conditions (e.g. luminal pH and bile acids) changes along the passage of digesta throughout the GIT [14]. Therefore, bacteria attached to the *Pars non-glandularis* of the stomach had the greatest nutrient availability which progressively degraded to the mid-colon. In the lower parts of the GIT, mainly highly complex non-starch polysaccharides, such as arabinoxylans and cellulose, remain in intestinal digesta, and mucus becomes a more important bacterial substrate. Interestingly, compared to the corn diets, wheat-barley diets promoted the colonic mucosal abundance of *Lactobacillus amylovorus* (OTU 2) which is involved in starch degradation in the upper GIT by 5.3% units [53]. It can be assumed that dietary starch was mostly digested until the distal ileum and proximal large intestine. Therefore, complex carbohydrates other than starch might have supported mucosal abundance of *L. amylovorus* and also of *Streptococcus alactolyticus* (OTU 11). It might be also thinkable that wheat-barley diets modified the glycocalix of the mucosa leading to a different microbe-host interaction benefiting certain phylotypes. Because of the predominance of *L. amylovorus* in the porcine GIT [17], information on its growth behavior *in vivo* is vital to characterize bacterial responses to differently composed diets [54]. By contrast, changes in the availability of complex hemicelluloses and cellulose likely enhanced mucosal abundance of *Prevotella stercorea* (OTU 49) in the colon with corn vs. wheat-barley diets [38].

Interestingly, bacterial genera that include potential opportunistic pathogens could be detected at all GIT sites (e.g. *Campylobacter*, *Fusobacteria* and *Clostridia*), representing a substantial proportion of the whole mucosa-associated community. Yet, all pigs included in our analyses did not show signs of enteric or systemic disease (data not shown). Similar observations were recently made in indoor versus outdoor reared piglets [19]. These findings indicate that a large pool of disease-associated species is commonly present in pig’s GIT awaiting critical changes in environmental conditions to become virulent.

In conclusion, the present data provided a comprehensive overview of the mucosa-associated microbiota in weaned pigs; thereby extending our understanding of the mucosal bacterial communities at different GIT sites. We reported a very high diversity of the microbiota attached to the *Pars non-glandularis* of the stomach which might be a sign for gastric health in young pigs. Accordingly, by enhancing attachment of *Lactobacillus* species to the gastric mucosa, Ca-P-rich diets may support gastric and overall gut health. Present data provide a fundamental basis for future research on diet-microbe-host interactions in weaned pigs.

## Supporting Information

Figure S1 **Microbial community similarity among all samples calculated with Bray-Curtis similarities.**
(PDF)

Figure S2 **pH-values in gastric, ileal and colonic digesta of weaned pigs.**
(PDF)

Figure S3 **Pairwise correlation analysis for the 20 most abundant OTUs per GIT site.**
(PDF)

Figure S4 **Phylogenetic relationship of Lactobacilli OTUs.**
(PDF)

Table S1 **Ingredients and analyzed chemical composition of the experimental diets.**
(PDF)

Table S2 **Barcoded primer sequences used in this study.**
(PDF)

Table S3 **Relative abundances of the 50 most abundant OTUs in the gastrointestinal sites independent of diet.**
(PDF)

Table S4 **Relative abundances of the 30 most abundant bacterial genera in the gastrointestinal sites independent of diet.**
(PDF)

Table S5 **Relative abundances of the 50 most abundant OTUs in the gastrointestinal tract of pigs fed wheat-barley or corn diets including adequate or high Ca-P.**
(PDF)

Table S6 **Relative abundances of the 30 most abundant bacterial genera in the gastrointestinal tract of pigs fed wheat-barley or corn diets including adequate or high Ca-P.**
(PDF)

Text S1 **Supplementary information about sequence processing and SAS procedures.**
(PDF)
