# Supplementary material for: Mucosa-Associated Bacterial Microbiome of the Gastrointestinal Tract of Weaned Pigs and Dynamics Linked to Dietary Calcium-Phosphorus
Source: PLoS One. 2014 Jan 23;9(1):e86950. doi: 10.1371/journal.pone.0086950 (PMC3900689; doi:10.1371/journal.pone.0086950)

Figure S1: Microbial community similarity among all samples. The heat map shows Bray-Curtis similarities based on abundances of OTUs (0.03 16S rRNA distance). Samples are listed according to GIT site and diet. Diets were abbreviated as follows: 1a) Wheat-barley diet with adequate Ca-P content; 1b) Wheat-barley diet with high Ca-P content; 2a) corn diet with adequate Ca-P content; 2b) Corn diet with high Ca-P content.

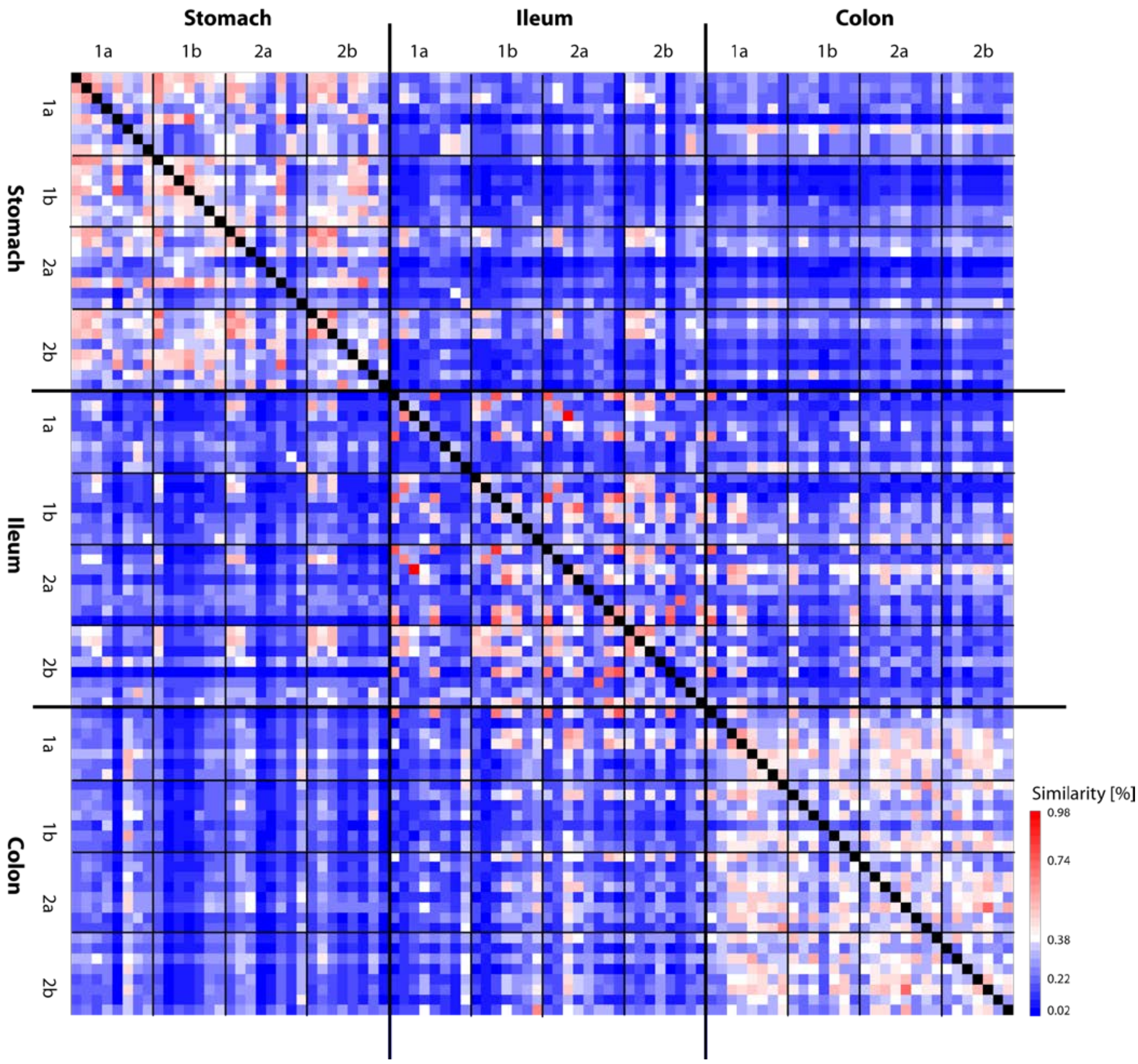

Supplement: Figure S1 — Microbial community similarity among all samples calculated with Bray-Curtis similarities. (PDF) [file pone.0086950.s001.pdf]
