# Supplementary figures and images for: Mucosa-Associated Bacterial Microbiome of the Gastrointestinal Tract of Weaned Pigs and Dynamics Linked to Dietary Calcium-Phosphorus
Source: PLoS One. 2014 Jan 23;9(1):e86950. doi: 10.1371/journal.pone.0086950 (PMC3900689; doi:10.1371/journal.pone.0086950)

**Supplemental Material Figure 2: pH-values in gastric, ileal and colonic digesta of weaned pigs.**

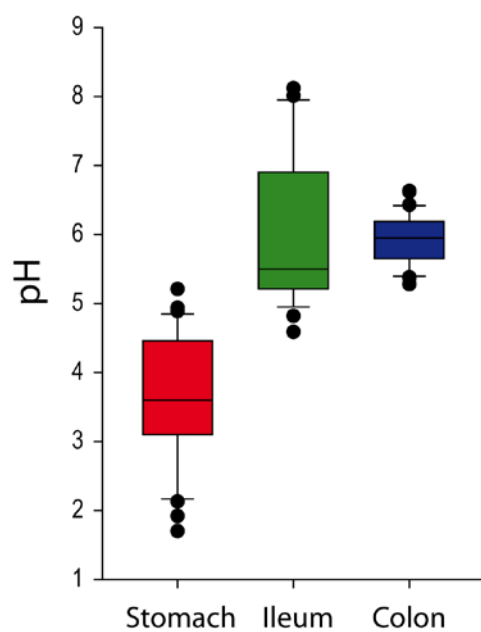

Supplement: Figure S2 — pH-values in gastric, ileal and colonic digesta of weaned pigs. (PDF) [file pone.0086950.s002.pdf]
