# Supplementary material for: Mucosa-Associated Bacterial Microbiome of the Gastrointestinal Tract of Weaned Pigs and Dynamics Linked to Dietary Calcium-Phosphorus
Source: PLoS One. 2014 Jan 23;9(1):e86950. doi: 10.1371/journal.pone.0086950 (PMC3900689; doi:10.1371/journal.pone.0086950)
Supplement: Figure S3 — Pairwise correlation analysis for the 20 most abundant OTUs per GIT site. (PDF) [file pone.0086950.s003.pdf]

Supplemental Material Figure 3: Pairwise correlation analysis for the 30 most abundant OTUs per GIT site, calculated with JMP Pro. The Pearson correlation coefficient (r) is calculated for each linear regression model. Dots represent values per sample and are colored as follows: Yellow: Wheat-barley diet with adequate Ca-P content; Orange: Wheat-barley diet with high Ca-P content; light blue: Corn diet with adequate Ca-P content; dark blue: Corn diet with high Ca-P content. Taxonomic classification of OTUs can be found in the manuscript (table 2). r>0.6 is highlighted yellowish.

Stomach

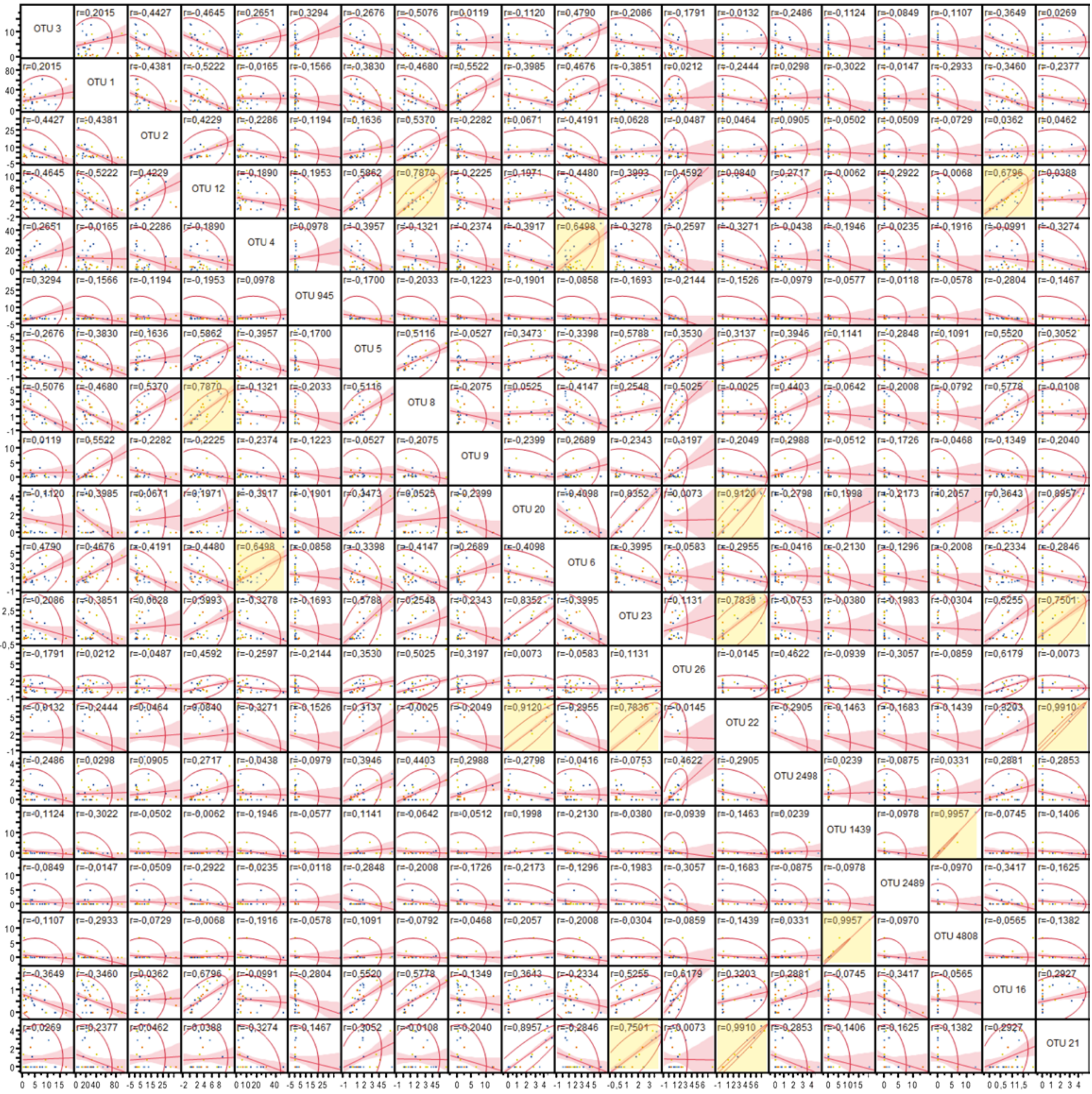

Ileum

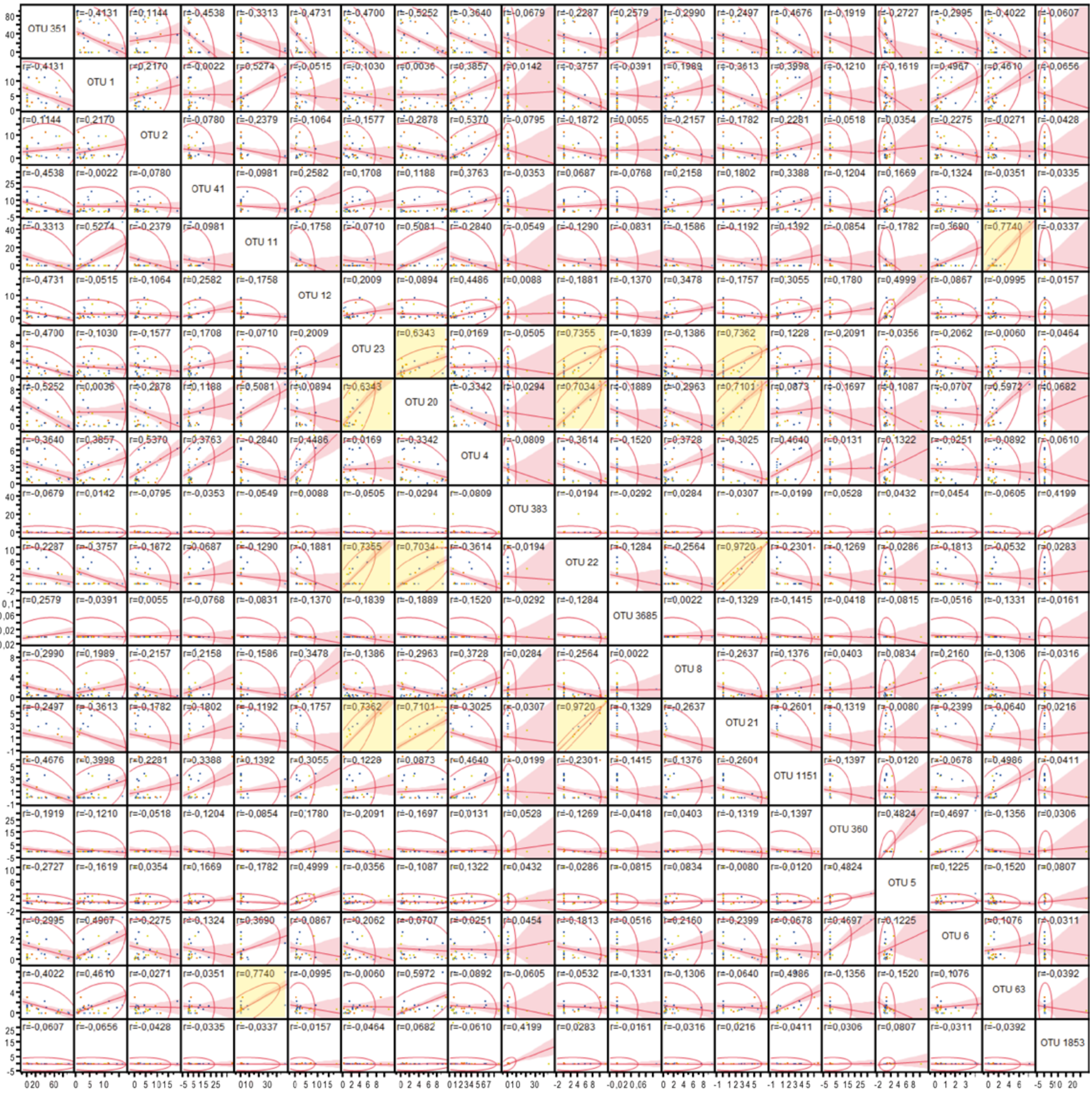
OTU 351 r=-0,4131 r=0,1144 r=-0,4538 r=-0,3313 r=-0,4731 r=-0,4700 r=-0,5252 r=-0,3640 r=-0,0679 r=-0,2287 r=0,2579 r=-0,2990 r=-0,2497 r=-0,4676 r=-0,1919 r=-0,2727 r=-0,2995 r=-0,4022 r=-0,0607
r=-0,4131 OTU 1 r=0,2170 r=-0,0022 r=0,5274 r=-0,0515 r=-0,1030 r=0,0036 r=0,3857 r=0,0142 r=-0,3757 r=-0,0391 r=0,1989 r=-0,3613 r=0,3998 r=-0,1210 r=-0,1619 r=0,4967 r=0,4610 r=-0,0656
r=0,1144 r=0,2170 OTU 2 r=-0,0780 r=-0,2379 r=-0,1064 r=-0,1577 r=-0,2878 r=0,5370 r=-0,0795 r=-0,1872 r=0,0055 r=-0,2157 r=-0,1782 r=0,2281 r=-0,0518 r=0,0354 r=-0,2275 r=-0,0271 r=-0,0428
r=-0,4538 r=-0,0022 r=-0,0780 OTU 41 r=-0,0981 r=0,2582 r=0,1708 r=0,1188 r=0,3763 r=-0,0353 r=0,0687 r=-0,0768 r=0,2158 r=0,1802 r=0,3388 r=-0,1204 r=0,1669 r=-0,1324 r=-0,0351 r=-0,0335
r=-0,3313 r=0,5274 r=-0,2379 r=-0,0981 OTU 11 r=-0,1758 r=-0,0710 r=0,5081 r=-0,2840 r=-0,0549 r=-0,1290 r=-0,0831 r=-0,1586 r=-0,1192 r=0,1392 r=-0,0854 r=-0,1782 r=0,3690 r=0,7740 r=-0,0337
r=-0,4731 r=-0,0515 r=-0,1064 r=0,2582 r=-0,1758 OTU 12 r=0,2009 r=-0,0894 r=0,4486 r=0,0088 r=-0,1881 r=-0,1370 r=0,3478 r=-0,1757 r=0,3055 r=0,1780 r=0,4999 r=-0,0867 r=-0,0995 r=-0,0157
r=-0,4700 r=-0,1030 r=-0,1577 r=0,1708 r=-0,0710 r=0,2009 OTU 23 r=0,6343 r=0,0169 r=-0,0505 r=0,7355 r=-0,1839 r=-0,1386 r=0,7362 r=0,1228 r=-0,2091 r=-0,0356 r=-0,2062 r=-0,0060 r=-0,0464
r=-0,5252 r=0,0036 r=-0,2878 r=0,1188 r=0,5081 r=-0,0894 r=0,6343 OTU 20 r=-0,3342 r=-0,0294 r=0,7034 r=-0,1889 r=-0,2963 r=0,7101 r=0,0873 r=-0,1697 r=-0,1087 r=-0,0707 r=0,5972 r=0,0682
r=-0,3640 r=0,3857 r=0,5370 r=0,3763 r=-0,2840 r=0,4486 r=0,0169 r=-0,3342 OTU 4 r=-0,0809 r=-0,3614 r=-0,1520 r=0,3728 r=-0,3025 r=0,4640 r=0,0131 r=0,1322 r=-0,0251 r=-0,0892 r=-0,0610
r=-0,0679 r=0,0142 r=-0,0795 r=-0,0353 r=-0,0549 r=0,0088 r=-0,0505 r=-0,0294 r=-0,0809 OTU 383 r=-0,0194 r=-0,0292 r=0,0284 r=-0,0307 r=-0,0199 r=0,0528 r=0,0432 r=0,0454 r=-0,0605 r=0,4199
r=-0,2287 r=-0,3757 r=-0,1872 r=0,0687 r=-0,1290 r=-0,1881 r=0,7355 r=0,7034 r=-0,3614 r=-0,0194 OTU 22 r=-0,1284 r=-0,2564 r=0,9720 r=-0,2301 r=-0,1269 r=-0,0286 r=-0,1813 r=-0,0532 r=0,0283
r=0,2579 r=-0,0391 r=0,0055 r=-0,0768 r=-0,0831 r=-0,1370 r=-0,1839 r=-0,1889 r=-0,1520 r=-0,0292 r=-0,1284 OTU 3685 r=0,0022 r=-0,1329 r=-0,1415 r=-0,0418 r=-0,0815 r=-0,0516 r=-0,1331 r=-0,0161
r=-0,2990 r=0,1989 r=-0,2157 r=0,2158 r=-0,1586 r=0,3478 r=-0,1386 r=-0,2963 r=0,3728 r=0,0284 r=-0,2564 r=0,0022 OTU 8 r=-0,2637 r=0,1376 r=0,0403 r=0,0834 r=0,2160 r=-0,1306 r=-0,0316
r=-0,2497 r=-0,3613 r=-0,1782 r=0,1802 r=-0,1192 r=-0,1757 r=0,7362 r=0,7101 r=-0,3025 r=-0,0307 r=0,9720 r=-0,1329 r=-0,2637 OTU 21 r=-0,2601 r=-0,1319 r=-0,0080 r=-0,2399 r=-0,0640 r=0,0216
r=-0,4676 r=0,3998 r=0,2281 r=0,3388 r=0,1392 r=0,3055 r=0,1228 r=0,0873 r=0,4640 r=-0,0199 r=-0,2301 r=-0,1415 r=0,1376 r=-0,2601 OTU 1151 r=-0,1397 r=-0,0120 r=-0,0678 r=0,4986 r=-0,0411
r=-0,1919 r=-0,1210 r=-0,0518 r=-0,1204 r=-0,0854 r=0,1780 r=-0,2091 r=-0,1697 r=0,0131 r=0,0528 r=-0,1269 r=-0,0418 r=0,0403 r=-0,1319 r=-0,1397 OTU 360 r=0,4824 r=0,4697 r=-0,1356 r=0,0306
r=-0,2727 r=-0,1619 r=0,0354 r=0,1669 r=-0,1782 r=0,4999 r=-0,0356 r=-0,1087 r=0,1322 r=0,0432 r=-0,0286 r=-0,0815 r=0,0834 r=-0,0080 r=-0,0120 r=0,4824 OTU 5 r=0,1225 r=-0,1520 r=0,0807
r=-0,2995 r=0,4967 r=-0,2275 r=-0,1324 r=0,3690 r=-0,0867 r=-0,2062 r=-0,0707 r=-0,0251 r=0,0454 r=-0,1813 r=-0,0516 r=0,2160 r=-0,2399 r=-0,0678 r=0,4697 r=0,1225 OTU 6 r=0,1076 r=-0,0311
r=-0,4022 r=0,4610 r=-0,0271 r=-0,0351 r=0,7740 r=-0,0995 r=-0,0060 r=0,5972 r=-0,0892 r=-0,0605 r=-0,0532 r=-0,1331 r=-0,1306 r=-0,0640 r=0,4986 r=-0,1356 r=-0,1520 r=0,1076 OTU 63 r=-0,0392
r=-0,0607 r=-0,0656 r=-0,0428 r=-0,0335 r=-0,0337 r=-0,0157 r=-0,0464 r=0,0682 r=-0,0610 r=0,4199 r=0,0283 r=-0,0161 r=-0,0316 r=0,0216 r=-0,0411 r=0,0306 r=0,0807 r=-0,0311 r=-0,0392 OTU 1853

Colon

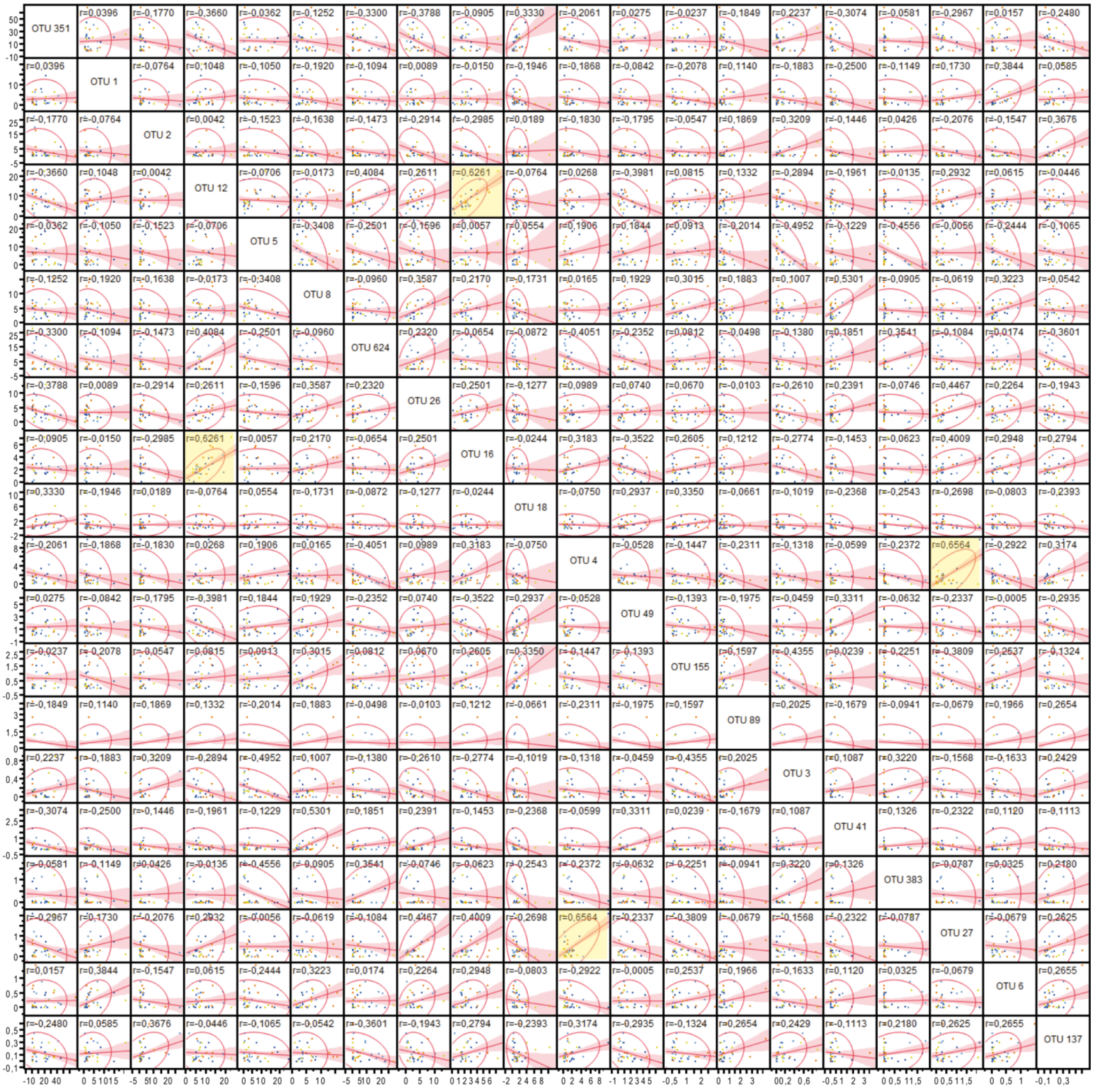

OTU 351 r=0,0396 r=-0,1770 r=-0,3660 r=-0,0362 r=-0,1252 r=-0,3300 r=-0,3788 r=-0,0905 r=0,3330 r=-0,2061 r=0,0275 r=-0,0237 r=-0,1849 r=0,2237 r=-0,3074 r=-0,0581 r=-0,2967 r=0,0157 r=-0,2480
r=0,0396 OTU 1 r=-0,0764 r=0,1048 r=-0,1050 r=-0,1920 r=-0,1094 r=0,0089 r=-0,0150 r=-0,1946 r=-0,1868 r=-0,0842 r=-0,2078 r=0,1140 r=-0,1883 r=-0,2500 r=-0,1149 r=0,1730 r=0,3844 r=0,0585
r=-0,1770 r=-0,0764 OTU 2 r=0,0042 r=-0,1523 r=-0,1638 r=-0,1473 r=-0,2914 r=-0,2985 r=0,0189 r=-0,1830 r=-0,1795 r=-0,0547 r=0,1869 r=0,3209 r=-0,1446 r=0,0426 r=-0,2076 r=-0,1547 r=0,3676
r=-0,3660 r=0,1048 r=0,0042 OTU 12 r=-0,0706 r=-0,0173 r=0,4084 r=0,2611 r=0,6261 r=-0,0764 r=0,0268 r=-0,3981 r=0,0815 r=0,1332 r=-0,2894 r=-0,1961 r=-0,0135 r=0,2932 r=0,0615 r=-0,0446
r=-0,0362 r=-0,1050 r=-0,1523 r=-0,0706 OTU 5 r=-0,3408 r=-0,2501 r=-0,1596 r=0,0057 r=0,0554 r=0,1906 r=0,1844 r=0,0913 r=-0,2014 r=-0,4952 r=-0,1229 r=-0,4556 r=-0,0056 r=-0,2444 r=-0,1065
r=-0,1252 r=-0,1920 r=-0,1638 r=-0,0173 r=-0,3408 OTU 8 r=-0,0960 r=0,3587 r=0,2170 r=-0,1731 r=0,0165 r=0,1929 r=0,3015 r=0,1883 r=0,1007 r=0,5301 r=-0,0905 r=-0,0619 r=0,3223 r=-0,0542
r=-0,3300 r=-0,1094 r=-0,1473 r=0,4084 r=-0,2501 r=-0,0960 OTU 624 r=0,2320 r=-0,0654 r=-0,0872 r=-0,4051 r=-0,2352 r=0,0812 r=-0,0498 r=-0,1380 r=0,1851 r=0,3541 r=-0,1084 r=0,0174 r=-0,3601
r=-0,3788 r=0,0089 r=-0,2914 r=0,2611 r=-0,1596 r=0,3587 r=0,2320 OTU 26 r=0,2501 r=-0,1277 r=0,0989 r=0,0740 r=0,0670 r=-0,0103 r=-0,2610 r=0,2391 r=-0,0746 r=0,4467 r=0,2264 r=-0,1943
r=-0,0905 r=-0,0150 r=-0,2985 r=0,6261 r=0,0057 r=0,2170 r=-0,0654 r=0,2501 OTU 16 r=-0,0244 r=0,3183 r=-0,3522 r=0,2605 r=0,1212 r=-0,2774 r=-0,1453 r=-0,0623 r=0,4009 r=0,2948 r=0,2794
r=0,3330 r=-0,1946 r=0,0189 r=-0,0764 r=0,0554 r=-0,1731 r=-0,0872 r=-0,1277 r=-0,0244 OTU 18 r=-0,0750 r=0,2937 r=0,3350 r=-0,0661 r=-0,1019 r=-0,2368 r=-0,2543 r=-0,2698 r=-0,0803 r=-0,2393
r=-0,2061 r=-0,1868 r=-0,1830 r=0,0268 r=0,1906 r=0,0165 r=-0,4051 r=0,0989 r=0,3183 r=-0,0750 OTU 4 r=-0,0528 r=-0,1447 r=-0,2311 r=-0,1318 r=-0,0599 r=-0,2372 r=0,6564 r=-0,2922 r=0,3174
r=0,0275 r=-0,0842 r=-0,1795 r=-0,3981 r=0,1844 r=0,1929 r=-0,2352 r=0,0740 r=-0,3522 r=0,2937 r=-0,0528 OTU 49 r=-0,1393 r=-0,1975 r=-0,0459 r=0,3311 r=-0,0632 r=-0,2337 r=-0,0005 r=-0,2935
r=-0,0237 r=-0,2078 r=-0,0547 r=0,0815 r=0,0913 r=0,3015 r=0,0812 r=0,0670 r=0,2605 r=0,3350 r=-0,1447 r=-0,1393 OTU 155 r=0,1597 r=-0,4355 r=0,0239 r=-0,2251 r=-0,3809 r=0,2537 r=-0,1324
r=-0,1849 r=0,1140 r=0,1869 r=0,1332 r=-0,2014 r=0,1883 r=-0,0498 r=-0,0103 r=0,1212 r=-0,0661 r=-0,2311 r=-0,1975 r=0,1597 OTU 89 r=0,2025 r=-0,1679 r=-0,0941 r=-0,0679 r=0,1966 r=0,2654
r=0,2237 r=-0,1883 r=0,3209 r=-0,2894 r=-0,4952 r=0,1007 r=-0,1380 r=-0,2610 r=-0,2774 r=-0,1019 r=-0,1318 r=-0,0459 r=-0,4355 r=0,2025 OTU 3 r=0,1087 r=0,3220 r=-0,1568 r=-0,1633 r=0,2429
r=-0,3074 r=-0,2500 r=-0,1446 r=-0,1961 r=-0,1229 r=0,5301 r=0,1851 r=0,2391 r=-0,1453 r=-0,2368 r=-0,0599 r=0,3311 r=0,0239 r=-0,1679 r=0,1087 OTU 41 r=0,1326 r=-0,2322 r=0,1120 r=-0,1113
r=-0,0581 r=-0,1149 r=0,0426 r=-0,0135 r=-0,4556 r=-0,0905 r=0,3541 r=-0,0746 r=-0,0623 r=-0,2543 r=-0,2372 r=-0,0632 r=-0,2251 r=-0,0941 r=0,3220 r=0,1326 OTU 383 r=-0,0787 r=0,0325 r=0,2180
r=-0,2967 r=0,1730 r=-0,2076 r=0,2932 r=-0,0056 r=-0,0619 r=-0,1084 r=0,4467 r=0,4009 r=-0,2698 r=0,6564 r=-0,2337 r=-0,3809 r=-0,0679 r=-0,1568 r=-0,2322 r=-0,0787 OTU 27 r=-0,0679 r=0,2625
r=0,0157 r=0,3844 r=-0,1547 r=0,0615 r=-0,2444 r=0,3223 r=0,0174 r=0,2264 r=0,2948 r=-0,0803 r=-0,2922 r=-0,0005 r=0,2537 r=0,1966 r=-0,1633 r=0,1120 r=0,0325 r=-0,0679 OTU 6 r=0,2655
r=-0,2480 r=0,0585 r=0,3676 r=-0,0446 r=-0,1065 r=-0,0542 r=-0,3601 r=-0,1943 r=0,2794 r=-0,2393 r=0,3174 r=-0,2935 r=-0,1324 r=0,2654 r=0,2429 r=-0,1113 r=0,2180 r=0,2625 r=0,2655 OTU 137
