# Supplementary material for: Mucosa-Associated Bacterial Microbiome of the Gastrointestinal Tract of Weaned Pigs and Dynamics Linked to Dietary Calcium-Phosphorus
Source: PLoS One. 2014 Jan 23;9(1):e86950. doi: 10.1371/journal.pone.0086950 (PMC3900689; doi:10.1371/journal.pone.0086950)

Supplemental Material Figure 4: Phylogenetic relationship of Lactobacilli OTUs. A 16S rRNA gene based RAxML maximum likelihood tree is shown. Three sequences from each *Lactobacillus* OTU were added to a bootstrapped (1000x) RAxML tree of full-length sequences using the quick-add parsimony tool without modifying the tree topology. All treeing was done with ARB. Sequences obtained in this study are shown in bold, GenBank accession numbers are indicated. Black dots indicate nodes with bootstrap support of at least 90%.

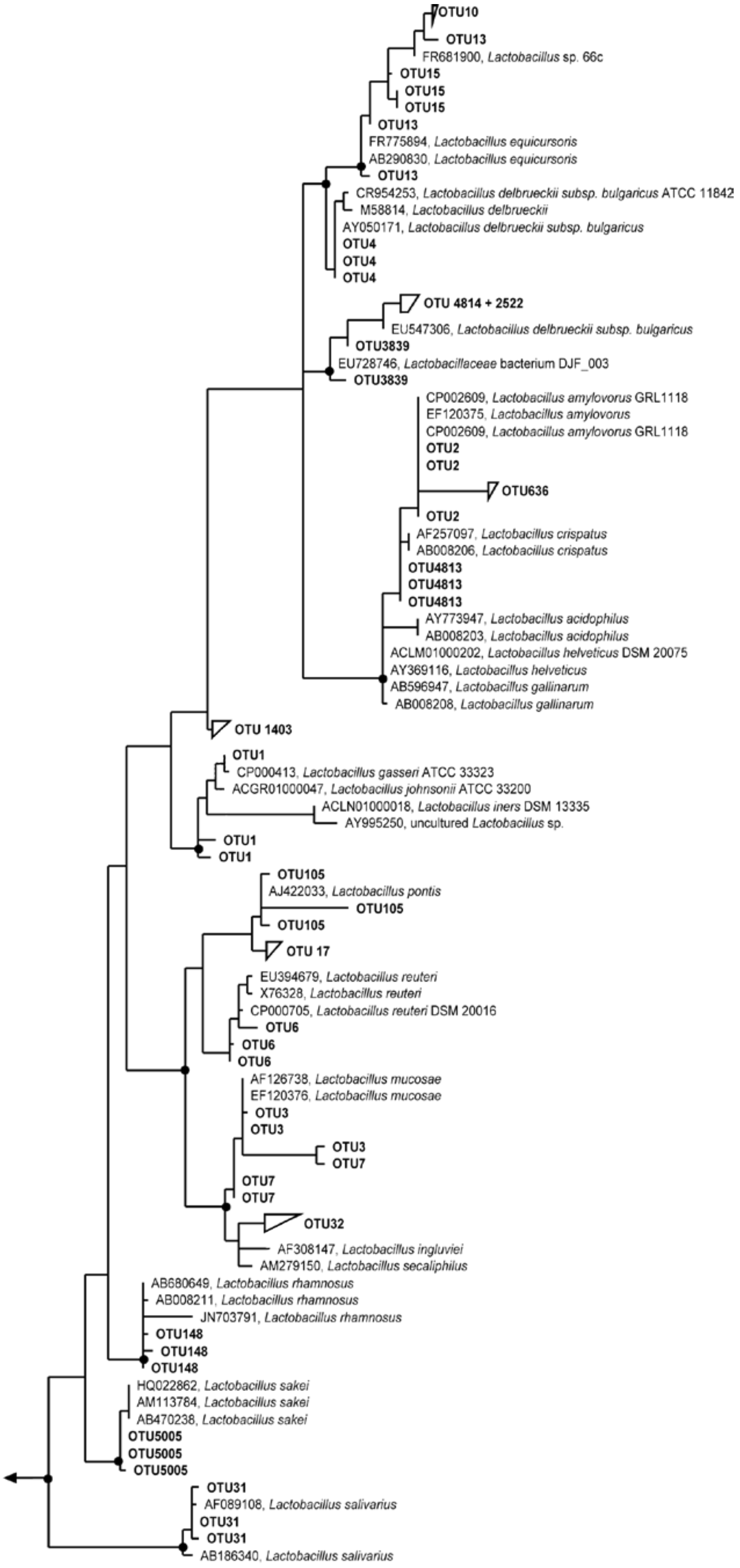

Supplement: Figure S4 — Phylogenetic relationship of Lactobacilli OTUs. (PDF) [file pone.0086950.s004.pdf]
