# Supplementary material for: Mucosa-Associated Bacterial Microbiome of the Gastrointestinal Tract of Weaned Pigs and Dynamics Linked to Dietary Calcium-Phosphorus
Source: PLoS One. 2014 Jan 23;9(1):e86950. doi: 10.1371/journal.pone.0086950 (PMC3900689; doi:10.1371/journal.pone.0086950)
Supplement: Table S1 — Ingredients and analyzed chemical composition of the experimental diets. (PDF) [file pone.0086950.s005.pdf]

Table S1. Ingredients and analyzed chemical composition of the experimental diets.

	Wheat-barley diet		Corn diet	
Ingredient (%)	Adequate Ca-P	High Ca-P	Adequate Ca-P	High Ca-P
Corn	-	-	49.40	49.40
Wheat	35.20	35.20	-	-
Soybean meal	28.00	28.00	33.50	33.50
Barley	18.20	18.20	-	-
Dextrose	5.00	5.00	5.00	5.00
Talkum	3.22	0.17	3.34	0.31
Saccharose	3.00	3.00	3.00	3.00
Soy oil	3.00	3.00	1.50	1.50
Limestone	1.30	1.95	1.25	1.93
Salt	1.00	1.00	1.00	1.00
Monocalcium phosphate	0.75	3.15	0.85	3.20
Vitamin-Mineral-Premix[a]	0.63	0.63	0.63	0.62
Lysine	0.35	0.35	0.24	0.25
L-Threonine	0.15	0.15	0.10	0.10
DL-Methionine	0.10	0.10	0.10	0.10
Phytase[b]	0.10	0.10	0.09	0.09
Analyzed chemical composition (dry matter basis)				
Dry matter (g kg^{-1})	929	917	921	929
Crude protein (g kg^{-1})	222	223	222	218
Crude ash (g kg^{-1})	94	96	95	96
Crude fiber (g kg^{-1})	36	37	36	36
Xylose (g kg^{-1})	16	18	11	12
β-Glucan (g kg^{-1})	9	9	1	1
NDF (g kg^{-1})	117	122	112	104
ADF (g kg^{-1})	85	79	83	68
Ca (g kg^{-1})	8.2	14.8	8.4	14.1
P (g kg^{-1})	6.0	11.9	6.6	11.7
ME (MJ/kg)[c]	15.11	15.15	15.22	15.27

Abbreviations: NDF, neutral detergent fiber; ADF, acid detergent fiber.

[a] Provided per kilogram of complete diet: 10,000 IU of vitamin A, 2,222 IU of vitamin D_3, 62.5 mg of vitamin E, 1.67 mg of vitamin B_1, 4.45 mg of vitamin B_2, 2.22 mg of vitamin B_6, 0.022 mg of vitamin B_{12}, 2.2 mg of vitamin K, 22.2 mg of niacin, 11.11 mg of pantothenic acid, 500 mg of choline chloride, 0.05 mg of biotin, 0.56 mg of folic acid, 25 mg of vitamin C; 44 mg of Mn (as MnO); 89 mg of Zn (as $ZnSO_4$); 153 mg of Fe (as $FeSO_4$), 13 mg of Cu (as $CuSO_4$), 0.44 mg of Se (as Na_2SeO_3), 1.67 mg of I (as $Ca(IO_3)_2$).

[b] Phytase activity, 500 FTU per kilogram of complete diet. Microbial phytase was added to the diets to compensate for the different intrinsic phytase activities in wheat, barley and corn.

[c] Calculated according to GfE (2006).
