# Supplementary material for: Mucosa-Associated Bacterial Microbiome of the Gastrointestinal Tract of Weaned Pigs and Dynamics Linked to Dietary Calcium-Phosphorus
Source: PLoS One. 2014 Jan 23;9(1):e86950. doi: 10.1371/journal.pone.0086950 (PMC3900689; doi:10.1371/journal.pone.0086950)
Supplement: Table S2 — Barcoded primer sequences used in this study. (PDF) [file pone.0086950.s006.pdf]

Table S2. Barcoded primer sequences used in this study. Amplicons were sequenced from the Titanium A adaptor (CCATCTCATCCCTGCGTGTCTCCGAC), followed by a four bases key sequence (TCAG) and the barcode followed by the target specific sequence. The reverse primer was used with the Titanium B adaptor (CCTATCCCCTGTGTGCCTTGGCAGTC) and the target specific sequence but without barcode sequence (CCTATCCCCTGTGTGCCTTGGCAGTC CTGCTGCCTYCCGTA).

Barcode ID	Barcode (5‘ - 3‘)	Target-specific sequence
1	ACGAGTGCGT	AGAGTTTGATCCTGGCTCAG
2	ACGCTCGACA	AGAGTTTGATCCTGGCTCAG
3	AGACGCACTC	AGAGTTTGATCCTGGCTCAG
4	AGCACTGTAG	AGAGTTTGATCCTGGCTCAG
5	ATCAGACACG	AGAGTTTGATCCTGGCTCAG
6	ATATCGCGAG	AGAGTTTGATCCTGGCTCAG
7	CGTGTCTCTA	AGAGTTTGATCCTGGCTCAG
8	CTCGCGTGTC	AGAGTTTGATCCTGGCTCAG
10	TCTCTATGCG	AGAGTTTGATCCTGGCTCAG
11	TGATACGTCT	AGAGTTTGATCCTGGCTCAG
13	CATAGTAGTG	AGAGTTTGATCCTGGCTCAG
14	CGAGAGATAC	AGAGTTTGATCCTGGCTCAG
15	ATACGACGTA	AGAGTTTGATCCTGGCTCAG
16	TCACGTACTA	AGAGTTTGATCCTGGCTCAG
17	CGTCTAGTAC	AGAGTTTGATCCTGGCTCAG
18	TCTACGTAGC	AGAGTTTGATCCTGGCTCAG
19	TGTACTACTC	AGAGTTTGATCCTGGCTCAG
20	ACGACTACAG	AGAGTTTGATCCTGGCTCAG
21	CGTAGACTAG	AGAGTTTGATCCTGGCTCAG
22	TACGAGTATG	AGAGTTTGATCCTGGCTCAG
23	TACTCTCGTG	AGAGTTTGATCCTGGCTCAG
24	TAGAGACGAG	AGAGTTTGATCCTGGCTCAG
25	TCGTCGCTCG	AGAGTTTGATCCTGGCTCAG
26	ACATACGCGT	AGAGTTTGATCCTGGCTCAG
27	ACGCGAGTAT	AGAGTTTGATCCTGGCTCAG
28	ACTACTATGT	AGAGTTTGATCCTGGCTCAG
29	ACTGTACAGT	AGAGTTTGATCCTGGCTCAG
30	AGACTATACT	AGAGTTTGATCCTGGCTCAG
31	AGCGTCGTCT	AGAGTTTGATCCTGGCTCAG
32	AGTACGCTAT	AGAGTTTGATCCTGGCTCAG
