# Supplementary material for: Mucosa-Associated Bacterial Microbiome of the Gastrointestinal Tract of Weaned Pigs and Dynamics Linked to Dietary Calcium-Phosphorus
Source: PLoS One. 2014 Jan 23;9(1):e86950. doi: 10.1371/journal.pone.0086950 (PMC3900689; doi:10.1371/journal.pone.0086950)
Supplement: Table S3 — Relative abundances of the 50 most abundant OTUs in the gastrointestinal sites independent of diet. (PDF) [file pone.0086950.s007.pdf]

Table S3. Relative abundances of the 50 most abundant OTUs in the gastrointestinal sites independent of diet. For stomach, ileum and colon mucosa the 50 most abundant bacterial OTUs, based on an OTU definition of 0.03 16S rRNA distance, are shown. Values are least squares and standard error of the mean (SEM). Statistically significant shifts are highlighted in orange, trends in pale orange.

		Relative abundance [%] and SEM values						
OTU no.	Taxonomy (genus level)	Stomach	SEM	Ileum	SEM	Colon	SEM	P-value
OTU 351	*Helicobacter*	0.2^{b}	3.89	29.6^{aA}	3.89	16.6^{aB}	3.99	<0.001
OTU 1	*Lactobacillus*	28.9^{a}	2.34	5.1^{b}	2.34	3.5^{b}	2.39	<0.001
OTU 2	*Lactobacillus*	6.6^{A}	1.30	3.8^{B}	1.30	4.1	1.323	0.135
OTU 12	*Prevotella*	3.2^{b}	0.65	2.8^{b}	0.65	8.6^{a}	0.66	<0.001
OTU 4	*Lactobacillus*	9.9^{a}	1.10	2.4^{b}	1.09	1.5^{b}	1.12	<0.001
OTU 5	*Prevotella*	1.6^{b}	0.71	1.0^{b}	0.71	6.0^{a}	0.73	<0.001
OTU 8	*Prevotella*	1.6^{b}	0.50	1.5^{b}	0.50	5.4^{a}	0.49	<0.001
OTU 20	*Escherichia*	1.3^{b}	0.37	3.0^{a}	0.37	0.1^{c}	0.38	<0.001
OTU 3	*Lactobacillus*	5.6^{a}	0.48	0.4^{b}	0.5	0.2^{b}	0.49	<0.001
OTU 41	*Clostridium*	0.3^{b}	0.82	4.3^{a}	0.82	0.3^{b}	0.84	0.001
OTU 23	*Pseudomonas*	1.0^{b}	0.30	2.8^{a}	0.30	$<0.1^{c}$	0.30	<0.001
OTU 624	*Campylobacter*	0.1^{b}	0.82	0.4^{b}	0.82	6.0^{a}	0.84	<0.001
OTU 3685	*Helicobacter*	<0.1	0.94	1.5	0.94	0.1	0.96	0.431
OTU 26	*Prevotella*	1.0^{b}	0.36	0.7^{b}	0.36	3.7^{a}	0.37	<0.001
OTU 22	*Bacteroides*	1.3^{aB}	0.40	2.3^{aA}	0.40	$<0.1^{b}$	0.40	0.001
OTU 2498	*Prevotella*	1.0^{a}	0.29	0.1^{b}	0.29	0.1^{b}	0.30	0.052
OTU 16	*Prevotella*	0.7^{b}	0.21	0.8^{b}	0.21	2.5^{a}	0.21	<0.001
OTU 11	*Streptococcus*	0.3^{b}	1.25	3.6^{aA}	1.25	$<0.1^{b}$	1.28	0.087
OTU 383	*Campylobacter*	$<0.1^{B}$	0.87	2.3^{A}	0.87	0.4	0.89	0.150
OTU 21	*Bacteroides*	0.7^{b}	0.23	1.3^{a}	0.23	$<0.1^{c}$	0.23	0.001
OTU 6	*Lactobacillus*	1.5^{a}	0.19	0.94^{b}	0.19	0.3^{c}	0.19	<0.001
OTU 1439	*Acinetobacter*	1.0	0.41	0.7	0.41	0.1	0.42	0.298
OTU 1853	*Clostridium*	<0.1	0.55	0.9	0.55	<0.1	0.56	0.407
OTU 4808	*Acinetobacter*	0.8^{A}	0.29	0.6	0.29	0.1^{B}	0.30	0.218
OTU 18	*Prevotella*	0.5^{b}	0.23	0.5^{b}	0.23	1.7^{a}	0.24	0.002
OTU 34	*Clostridium*	0.5^{aB}	0.20	0.9^{aA}	0.16	$<0.1^{b}$	0.16	0.002
OTU 2489	*Prevotella*	1.0^{a}	0.29	0.1^{b}	0.29	0.1^{b}	0.30	0.052
OTU 52	*Fusobacterium*	0.5^{b}	0.14	0.8^{a}	0.14	$<0.1^{c}$	0.15	0.0008
OTU 4692	*Acinetobacter*	0.6^{A}	0.23	0.4	0.23	$<0.1^{B}$	0.23	0.231
OTU 1151	*Citrobacter*	0.3^{b}	0.19	1.2^{a}	0.19	$<0.1^{b}$	0.19	<0.001
OTU 14	*Clostridium*	0.5^{a}	0.13	0.7^{a}	0.13	$<0.1^{b}$	0.13	0.001
OTU 95	*Prevotella*	0.4^{b}	0.10	0.3^{b}	0.10	1.0^{a}	0.10	<0.001
OTU 35	*Haemophilus*	0.4^{b}	0.14	0.8^{a}	0.14	$<0.1^{c}$	0.14	<0.001
OTU 49	*Prevotella*	0.3^{b}	0.15	0.1^{b}	0.15	1.4^{a}	0.15	<.0001
OTU 63	*Haemophilus*	0.3^{b}	0.16	0.9^{a}	0.16	$<0.1^{b}$	0.16	0.001
OTU 945	*Prevotella*	1.7	0.73	<0.1	0.74	<0.1	0.76	0.192
OTU 29	*Proteus*	0.4^{aB}	0.11	0.6^{aA}	0.11	$<0.1^{b}$	0.11	0.001
OTU 27	*Lachnospira*	0.4	0.08	0.4	0.08	0.4	0.08	0.872
OTU 056	*Acidovorax*	0.4^{aA}	0.13	0.6^{a}	0.13	$<0.1^{bB}$	0.14	0.011
OTU 360	*Bacteroides*	<0.1	0.62	1.2	0.62	<0.1	0.63	0.328
OTU 4809	*Acinetobacter*	0.4^{A}	0.16	0.2	0.16	$<0.1^{B}$	0.16	0.227
OTU 155	*Prevotella*	0.2^{b}	0.10	0.2^{b}	0.10	0.9^{a}	0.10	<.001
OTU 24	*Clostridium*	0.3^{B}	0.08	0.4^{aA}	0.08	$<0.1^{bA}$	0.08	0.001
OTU 51	*Streptococcus*	0.3^{A}	0.10	0.2	0.10	$<0.1^{B}$	0.10	0.129
OTU 137	*Faecalibacterium*	0.2	0.28	0.6	0.28	0.1	0.29	0.416
OTU 89	*Eubacterium*	0.2^{b}	0.10	0.1^{b}	0.10	0.8^{a}	0.10	<0.001
OTU 36	*Clostridium*	0.2^{bA}	0.07	0.5^{a}	0.07	$<0.1^{bB}$	0.07	<0.001
OTU 59	*Staphylococcus*	0.2^{a}	0.08	0.4^{a}	0.08	$<0.1^{b}$	0.08	0.003
OTU 7	*Lactobacillus*	0.4^{A}	0.13	0.4	0.13	0.1^{B}	0.13	0.183

OTU 30	*Xylanibacter*	0.3^{B}	0.09	0.1^{b}	0.09	0.6^{aA}	0.10		0.009

^{abc} values within a row marked with different lower case letters are significantly different ($P \leq 0.05$).

^{ABC} values within a row marked with different upper case letters within a row indicate a trend ($P \leq 0.10$)
