# Supplementary material for: Mucosa-Associated Bacterial Microbiome of the Gastrointestinal Tract of Weaned Pigs and Dynamics Linked to Dietary Calcium-Phosphorus
Source: PLoS One. 2014 Jan 23;9(1):e86950. doi: 10.1371/journal.pone.0086950 (PMC3900689; doi:10.1371/journal.pone.0086950)
Supplement: Table S4 — Relative abundances of the 30 most abundant bacterial genera in the gastrointestinal sites independent of diet. (PDF) [file pone.0086950.s008.pdf]

Table S4. Relative abundances of the 30 most abundant bacterial genera in the gastrointestinal sites independent of diet. For classification into genera, OTUs affiliating to the same genus were combined. Values are least squares and standard error of the mean (SEM). Statistically significant shifts are highlighted in orange, trends in pale orange.

	Relative abundance [%] and SEM values						
Taxonomy	Stomach	SEM	Ileum	SEM	Colon	SEM	P-value
Lactobacillus	54.5^{a}	2.70	13.4^{b}	2.70	9.7^{b}	2.77	<0.001
Prevotella	15.8^{b}	2.32	10.2^{c}	2.32	41.4^{a}	2.38	<0.001
Helicobacter	1.8^{c}	4.01	29.7^{a}	4.02	16.8^{b}	4.11	<0.001
Bacteroides	2.1^{aA}	0.89	5.0^{a}	0.89	<0.1^{bB}	0.91	0.001
Campylobacter	0.2^{b}	1.27	2.8^{b}	1.27	6.9^{a}	1.30	0.002
Clostridium XI	1.0^{b}	0.83	5.8^{a}	0.82	0.4^{b}	0.85	<0.001
Acinetobacter	3.1	1.23	2.0	1.23	0.2	1.26	0.252
Paraprevotella	1.3^{b}	0.66	1.1^{b}	0.66	5.2^{a}	0.67	<0.001
Pseudomonas	1.4^{b}	0.34	3.0^{a}	0.34	<0.1^{c}	0.35	<0.001
Escherichia-Shigella	1.3^{b}	0.37	3.0^{a}	0.37	0.1^{c}	0.38	<0.001
Streptococcus	0.7^{aA}	1.32	4.1^{aB}	1.32	<0.1^{b}	1.35	0.073
Lachnospiracea incertae sedis	0.6	0.10	0.5	0.10	0.7	0.10	0.280
Clostridium sensu stricto	0.7^{b}	0.17	1.4^{a}	0.17	0.1^{c}	0.17	<0.001
Faecalibacterium	0.6	0.30	1.1	0.30	0.6	0.31	0.355
Clostridium XIX	<0.1	0.55	0.9	0.55	<0.1	0.56	0.407
Haemophilus	0.7^{b}	0.15	1.1^{a}	0.15	<0.1^{c}	0.16	<0.001
Aminiphilus	0.5	0.12	0.7	0.12	0.5	0.12	0.454
Xylanibacter	0.4^{b}	0.15	0.4^{b}	0.15	1.5^{a}	0.16	<0.001
Fusobacterium	0.5^{aB}	0.14	0.8^{aA}	0.14	<0.1^{b}	0.15	<0.001
Citrobacter	0.3^{a}	0.19	1.2^{a}	0.19	<0.1^{b}	0.19	<0.001
Coprobacillus	0.5^{a}	0.13	0.7^{a}	0.13	<0.1^{b}	0.13	<0.001
Dialister	0.7^{a}	0.15	0.3^{b}	0.15	0.4^{ab}	0.16	0.077
Klebsiella	0.3^{a}	0.16	0.9^{b}	0.16	<0.1^{a}	0.16	<0.001
Proteus	0.4^{aA}	0.11	0.6^{aB}	0.11	<0.1^{b}	0.11	0.001
Oscillibacter	0.4	0.07	0.3	0.07	0.5	0.07	0.422
Phocaeicola	0.3^{b}	0.13	0.3^{b}	0.13	0.9^{a}	0.14	<0.001
Acidovorax	0.4^{A}	0.13	0.6^{a}	0.13	0.1^{bB}	0.14	0.011
Asteroleplasma	0.2^{b}	0.14	0.3^{b}	0.14	0.9^{a}	0.14	<0.001
Sediminitomix	0.2^{b}	0.12	0.1^{b}	0.12	0.9^{a}	0.12	<.0001
Rikenella	0.3^{b}	0.17	0.3^{b}	0.16	1.0^{a}	0.17	0.003

^{abc} values within a row marked with different lower case letters are significantly different ($P \leq 0.05$).

^{ABC} values within a row marked with different upper case letters within a row indicate a trend ($P \leq 0.10$).
