# Supplementary material for: Mucosa-Associated Bacterial Microbiome of the Gastrointestinal Tract of Weaned Pigs and Dynamics Linked to Dietary Calcium-Phosphorus
Source: PLoS One. 2014 Jan 23;9(1):e86950. doi: 10.1371/journal.pone.0086950 (PMC3900689; doi:10.1371/journal.pone.0086950)
Supplement: Table S5 — Relative abundances of the 50 most abundant OTUs in the gastrointestinal tract of pigs fed wheat-barley or corn diets including adequate or high Ca-P. (PDF) [file pone.0086950.s009.pdf]

Table S5. Relative abundances of the 50 most abundant OTUs in the gastrointestinal tract of pigs fed wheat-barley or corn diets including adequate or high Ca-P. OTUs are based on an OTU definition of 0.03 16S rRNA distance. Values are least squares and standard error of the mean (SEM). Statistically significant shifts are highlighted in orange, trends in pale orange.

OTU no.	Taxonomy (genus level)	Relative abundance [%]					*P*-Value		
		Wheat-Barley		Corn					
		Adequate Ca-P	High Ca-P	Adequate Ca-P	High Ca-P	SEM	Cereals	Ca-P	Cereals × Ca-P
**Stomach**									
OTU 351	*Helicobacter*	0.1	0.4	0.2	0.1	0.17	0.548	0.747	0.166
OTU 1	*Lactobacillus*	33.0	36.6	18.6	26.2	7.60	0.113	0.471	0.791
OTU 2	*Lactobacillus*	7.9	7.2	4.3	7.0	3.68	0.612	0.795	0.656
OTU 12	*Prevotella*	4.5	2.1	3.2	3.0	1.01	0.827	0.196	0.300
OTU 4	*Lactobacillus*	4.6	10.0	9.0	16.6	3.64	0.143	0.086	0.750
OTU 5	*Prevotella*	2.6	0.8	1.7	1.1	0.54	0.579	0.035	0.224
OTU 8	*Prevotella*	2.2	1.1	1.9	1.3	0.59	0.999	0.159	0.659
OTU 20	*Escherichia*	1.4	0.8	1.6	1.6	0.56	0.425	0.599	0.602
OTU 3	*Lactobacillus*	2.3	9.0	3.6	7.3	1.62	0.886	0.004	0.348
OTU 41	*Clostridium*	0.6	<0.1	0.2	0.4	0.21	0.955	0.412	0.072
OTU 23	*Pseudomonas*	1.1	0.6	1.3	1.0	0.42	0.515	0.301	0.854
OTU 624	*Campylobacter*	0.2	<0.0	0.1	<0.1	0.07	0.743	0.131	0.742
OTU 3685	*Helicobacter*	<0.1	<0.1	6.2	<0.1	3.26	0.327	0.327	0.365
OTU 26	*Prevotella*	1.9	0.4	1.1	0.8	0.53	0.723	0.128	0.278
OTU 22	*Bacteroides*	1.3	0.5	1.4	1.9	0.76	0.346	0.862	0.402
OTU 2498	*Prevotella*	1.1	0.4	1.1	0.6	0.42	0.764	0.168	0.906
OTU 16	*Prevotella*	1.0	0.4	0.7	0.6	0.18	0.541	0.077	0.172
OTU 11	*Streptococcus*	0.3	0.1	0.3	0.4	0.18	0.502	0.722	0.364
OTU 383	*Campylobacter*	<0.1	<0.1	<0.1	<0.1	0.02	0.076	0.923	0.870
OTU 21	*Bacteroides*	0.8	0.2	0.9	1.1	0.43	0.253	0.705	0.345
OTU 6	*Lactobacillus*	2.1	1.0	0.8	2.2	0.46	0.895	0.734	0.009
OTU 1439	*Acinetobacter*	0.9	0.5	2.6	0.1	1.26	0.589	0.255	0.418
OTU 1853	*Clostridium*	<0.1	<0.1	<0.1	<0.1	0.01	0.202	0.619	0.690
OTU 4808	*Acinetobacter*	1.1	0.4	1.7	0.1	0.86	0.897	0.182	0.596
OTU 18	*Prevotella*	0.7	0.5	0.5	0.2	0.14	0.070	0.138	0.733
OTU 34	*Clostridium*	0.5	0.2	0.5	0.8	0.32	0.445	0.895	0.358
OTU 2489	*Prevotella*	<0.1	1.8	1.4	0.8	0.92	0.858	0.510	0.208
OTU 52	*Fusobacterium*	0.4	0.3	0.4	0.7	0.24	0.313	0.675	0.361
OTU 4692	*Acinetobacter*	0.7	0.3	1.4	0.1	0.67	0.783	0.204	0.524
OTU 1151	*Citrobacter*	0.5	0.3	0.3	0.1	0.12	0.201	0.107	0.785
OTU 14	*Clostridium*	0.4	0.3	0.4	0.7	0.25	0.454	0.734	0.352
OTU 95	*Prevotella*	0.6	0.3	0.4	0.2	0.15	0.425	0.162	0.723
OTU 35	*Haemophilus*	0.4	0.1	0.4	0.7	0.23	0.139	0.881	0.228
OTU 49	*Prevotella*	0.6	0.1	0.3	0.2	0.15	0.458	0.060	0.207
OTU 63	*Haemophilus*	0.3	0.2	0.3	0.4	0.13	0.447	0.879	0.810
OTU 945	*Prevotella*	<0.1	0.8	5.7	0.2	2.56	0.324	0.363	0.235
OTU 29	*Proteus*	0.3	0.2	0.4	0.6	0.21	0.286	0.899	0.410
OTU 27	*Lachnospira*	0.7	0.2	0.3	0.3	0.16	0.438	0.093	0.162
OTU 056	*Acidovorax*	0.3	0.5	0.6	0.2	0.22	0.951	0.581	0.161
OTU 360	*Bacteroides*	<0.1	<0.1	<0.1	<0.1	0.02	0.520	0.525	0.220
OTU 4809	*Acinetobacter*	0.6	0.2	0.9	<0.1	0.48	0.883	0.218	0.572
OTU 155	*Prevotella*	0.3	0.2	0.1	0.1	0.09	0.130	0.330	0.349
OTU 24	*Clostridium*	0.3	0.2	0.2	0.4	0.18	0.763	0.746	0.411
OTU 51	*Streptococcus*	0.2	0.4	0.2	0.3	0.15	0.870	0.342	0.759
OTU 137	*Faecalibacterium*	0.2	0.1	0.2	0.2	0.05	0.467	0.650	0.451
OTU 89	*Eubacterium*	0.3	0.1	0.2	0.2	0.11	0.825	0.379	0.482

OTU 36	*Clostridium*	0.1	0.1		0.2	0.3		0.10		0.102	0.982	0.433
OTU 59	*Staphylococcus*	0.2	0.1		0.2	0.5		0.14		0.179	0.630	0.132
OTU 7	*Lactobacillus*	0.6	0.8		0.1	0.3		0.32		0.121	0.497	0.951
OTU 30	*Xylanibacter*	0.3	0.1		0.5	0.5		0.10		0.059	0.488	0.475
**Ileum**												
OTU 351	*Helicobacter*	22.6	29.2		41.9	24.9		12.33		0.547	0.678	0.345
OTU 1	*Lactobacillus*	5.6	4.0		4.7	6.8		1.61		0.540	0.887	0.258
OTU 2	*Lactobacillus*	3.5	4.6		2.9	4.3		1.61		0.788	0.468	0.927
OTU 12	*Prevotella*	4.8	2.4		1.8	2.1		1.26		0.203	0.401	0.290
OTU 4	*Lactobacillus*	2.1	2.0		1.8	3.5		0.79		0.457	0.288	0.266
OTU 5	*Prevotella*	2.2	0.7		0.7	0.6		0.66		0.216	0.225	0.290
OTU 8	*Prevotella*	1.5	1.0		2.1	1.6		0.79		0.464	0.520	0.981
OTU 20	*Escherichia*	2.8	3.1		2.0	3.8		1.05		0.946	0.298	0.457
OTU 3	*Lactobacillus*	0.2	0.4		0.4	0.8		0.10		0.072	0.078	0.519
OTU 41	*Clostridium*	1.4	7.3		1.5	6.9		2.86		0.949	0.058	0.935
OTU 23	*Pseudomonas*	3.6	3.3		1.8	2.7		0.88		0.191	0.754	0.504
OTU 624	*Campylobacter*	0.6	0.7		0.1	0.1		0.37		0.139	0.972	0.821
OTU 3685	*Helicobacter*	<0.1	<0.1		<0.1	<0.1		0.01		0.327	0.327	0.365
OTU 26	*Prevotella*	1.0	0.5		0.7	0.5		0.36		0.681	0.323	0.692
OTU 22	*Bacteroides*	2.3	3.1		1.6	2.2		0.88		0.364	0.441	0.921
OTU 2498	*Prevotella*	0.6	0.4		1.0	0.3		0.38		0.750	0.272	0.492
OTU 16	*Prevotella*	0.9	0.6		1.0	0.6		0.39		0.928	0.376	0.935
OTU 11	*Streptococcus*	0.1	<0.0		7.0	7.3		4.16		0.094	0.996	0.945
OTU 383	*Campylobacter*	8.4	0.3		0.2	0.1		2.88		0.159	0.160	0.176
OTU 21	*Bacteroides*	1.3	1.6		1.0	1.4		0.45		0.535	0.458	0.982
OTU 6	*Lactobacillus*	0.8	0.7		1.2	1.1		0.41		0.321	0.784	0.908
OTU 1439	*Acinetobacter*	1.2	0.4		0.1	1.1		0.61		0.739	0.806	0.151
OTU 1853	*Clostridium*	3.6	<0.1		0.1	<0.1		1.90		0.365	0.327	0.341
OTU 4808	*Acinetobacter*	1.1	0.4		<0.1	0.7		0.49		0.453	0.934	0.165
OTU 18	*Prevotella*	0.4	0.8		0.5	0.3		0.29		0.536	0.678	0.278
OTU 34	*Clostridium*	1.0	1.2		0.5	0.8		0.36		0.291	0.456	0.865
OTU 2489	*Prevotella*	<0.1	0.1		0.1	0.1		0.07		0.694	0.774	0.216
OTU 52	*Fusobacterium*	0.9	1.2		0.5	0.7		0.34		0.265	0.451	0.932
OTU 4692	*Acinetobacter*	0.9	0.3		<0.1	0.5		0.37		0.380	0.842	0.145
OTU 1151	*Citrobacter*	0.7	2.2		0.1	1.7		0.62		0.378	0.016	0.908
OTU 14	*Clostridium*	0.7	0.9		0.4	0.8		0.28		0.493	0.338	0.615
OTU 95	*Prevotella*	0.6	0.2		0.3	0.3		0.16		0.687	0.166	0.262
OTU 35	*Haemophilus*	0.7	1.2		0.6	0.8		0.33		0.501	0.393	0.636
OTU 49	*Prevotella*	<0.1	0.1		0.2	0.2		0.06		0.010	0.826	0.982
OTU 63	*Haemophilus*	0.5	0.9		0.4	2.0		0.53		0.359	0.061	0.256
OTU 945	*Prevotella*	<0.1	0.1		0.1	<0.1		0.04		0.388	0.886	0.335
OTU 29	*Proteus*	0.6	0.9		0.4	0.5		0.24		0.252	0.483	0.782
OTU 27	*Lachnospira*	0.4	0.4		0.3	0.4		0.13		0.581	0.729	0.519
OTU 056	*Acidovorax*	0.7	1.1		0.4	0.2		0.38		0.150	0.891	0.488
OTU 360	*Bacteroides*	0.3	0.1		4.1	<0.1		2.15		0.379	0.323	0.372
OTU 4809	*Acinetobacter*	0.5	0.1		<0.1	0.1		0.22		0.304	0.524	0.268
OTU 155	*Prevotella*	0.3	0.1		0.1	0.1		0.11		0.394	0.215	0.315
OTU 24	*Clostridium*	0.4	0.7		0.3	0.4		0.17		0.170	0.261	0.648
OTU 51	*Streptococcus*	0.6	0.1		<0.1	0.2		0.30		0.487	0.637	0.255
OTU 137	*Faecalibacterium*	0.2	0.1		1.9	0.2		0.96		0.332	0.357	0.395
OTU 89	*Eubacterium*	0.1	0.1		0.1	0.2		0.06		0.765	0.333	0.219
OTU 36	*Clostridium*	0.5	0.7		0.4	0.4		0.17		0.207	0.408	0.643
OTU 59	*Staphylococcus*	0.5	0.5		0.3	0.4		0.21		0.627	0.748	0.729
OTU 7	*Lactobacillus*	<0.1	0.7		0.5	0.3		0.25		0.849	0.326	0.101
OTU 30	*Xylanibacter*	<0.1	0.1		0.3	0.1		0.01		0.432	0.516	0.213
**Colon**												
OTU 351	*Helicobacter*	22.4	12.2		17.6	13.7		6.60		0.802	0.293	0.633
OTU 1	*Lactobacillus*	3.9	1.5		3.9	4.9		1.83		0.352	0.714	0.365

OTU 2	*Lactobacillus*	4.4	9.4		1.6	1.2		2.39		0.030	0.347	0.273
OTU 12	*Prevotella*	9.3	6.5		11.6	7.4		1.70		0.353	0.050	0.681
OTU 4	*Lactobacillus*	0.9	0.8		1.8	2.1		0.72		0.133	0.905	0.750
OTU 5	*Prevotella*	4.8	9.4		4.1	6.0		2.48		0.414	0.199	0.576
OTU 8	*Prevotella*	5.5	6.0		3.6	6.1		1.34		0.475	0.265	0.455
OTU 20	*Escherichia*	0.1	0.1		0.1	0.1		0.04		0.413	0.395	0.777
OTU 3	*Lactobacillus*	0.1	0.2		0.2	0.3		0.00		0.185	0.397	0.880
OTU 41	*Clostridium*	0.5	0.2		0.1	0.5		0.00		0.918	0.861	0.254
OTU 23	*Pseudomonas*	<0.1	<0.1		<0.1	<0.1		0.01		0.928	0.922	0.832
OTU 624	*Campylobacter*	8.3	3.4		9.0	3.0		2.95		0.966	0.072	0.861
OTU 3685	*Helicobacter*	<0.1	<0.1		<0.1	<0.1		0.00		-	-	-
OTU 26	*Prevotella*	2.3	3.9		4.1	4.2		1.11		0.359	0.429	0.480
OTU 22	*Bacteroides*	0.1	<0.1		<0.1	<0.1		0.02		0.148	0.065	0.258
OTU 2498	*Prevotella*	4.5	3.1		4.2	3.2		1.25		0.959	0.345	0.897
OTU 16	*Prevotella*	2.9	1.9		3.2	2.2		0.63		0.657	0.120	0.968
OTU 11	*Streptococcus*	<0.1	<0.1		0.1	0.1		0.05		0.036	0.871	0.976
OTU 383	*Campylobacter*	0.2	0.2		0.7	0.1		0.19		0.261	0.131	0.158
OTU 21	*Bacteroides*	0.1	<0.1		<0.1	<0.1		0.03		0.741	0.181	0.369
OTU 6	*Lactobacillus*	0.4	0.2		0.3	0.2		0.12		0.289	0.206	0.630
OTU 1439	*Acinetobacter*	<0.1	<0.1		<0.1	<0.1		0.01		0.541	0.495	0.322
OTU 1853	*Clostridium*	<0.1	<0.1		<0.1	<0.1		0.01		0.272	0.272	0.223
OTU 4808	*Acinetobacter*	<0.1	<0.1		<0.1	<0.1		0.01		0.895	0.223	0.705
OTU 18	*Prevotella*	1.2	1.4		1.5	2.6		0.78		0.308	0.404	0.564
OTU 34	*Clostridium*	<0.1	<0.1		<0.1	<0.1		0.01		0.624	0.465	0.627
OTU 2489	*Prevotella*	0.1	<0.1		<0.1	<0.1		0.07		0.347	0.322	0.456
OTU 52	*Fusobacterium*	<0.1	<0.1		<0.1	<0.1		0.01		0.312	0.475	0.475
OTU 4692	*Acinetobacter*	<0.1	<0.1		<0.1	<0.1		0.01		0.083	0.680	0.443
OTU 1151	*Citrobacter*	<0.1	<0.1		<0.1	0.1		0.02		0.922	0.228	0.488
OTU 14	*Clostridium*	<0.1	<0.1		<0.1	<0.1		0.01		0.454	0.689	0.620
OTU 95	*Prevotella*	1.0	0.7		1.0	1.2		0.29		0.399	0.921	0.356
OTU 35	*Haemophilus*	<0.1	<0.1		<0.1	<0.1		0.01		0.127	0.614	0.499
OTU 49	*Prevotella*	0.8	1.0		1.3	2.8		0.52		0.038	0.107	0.242
OTU 63	*Haemophilus*	<0.1	<0.1		<0.1	<0.1		0.01		0.092	0.742	0.653
OTU 945	*Prevotella*	0.1	<0.1		<0.1	<0.1		0.02		0.258	0.293	0.155
OTU 29	*Proteus*	<0.1	<0.1		<0.1	<0.1		0.01		0.224	0.509	0.991
OTU 27	*Lachnospira*	0.4	0.4		0.7	0.3		0.22		0.641	0.379	0.450
OTU 056	*Acidovorax*	<0.1	<0.1		<0.1	<0.1		0.01		0.224	0.196	0.973
OTU 360	*Bacteroides*	<0.1	<0.1		<0.1	<0.1		0.00		0.326	0.326	0.412
OTU 4809	*Acinetobacter*	<0.1	<0.1		<0.1	<0.1		0.00		0.409	0.409	0.323
OTU 155	*Prevotella*	0.9	1.3		0.5	0.7		0.31		0.157	0.340	0.763
OTU 24	*Clostridium*	<0.1	<0.1		<0.1	<0.1		0.01		0.973	0.273	0.896
OTU 51	*Streptococcus*	<0.1	<0.1		<0.1	<0.1		0.00		0.247	0.247	0.247
OTU 137	*Faecalibacterium*	0.1	0.2		0.2	0.1		0.06		0.935	0.703	0.530
OTU 89	*Eubacterium*	0.7	1.2		0.5	0.7		0.33		0.286	0.199	0.665
OTU 36	*Clostridium*	<0.1	<0.1		<0.1	<0.1		0.01		0.420	0.537	0.056
OTU 59	*Staphylococcus*	<0.1	<0.1		<0.1	<0.1		0.01		0.281	0.859	0.977
OTU 7	*Lactobacillus*	0.3	<0.1		0.1	0.1		0.15		0.599	0.384	0.430
OTU 30	*Xylanibacter*	0.9	0.2		0.5	0.7		0.27		0.740	0.337	0.174
