# Supplementary material for: Mucosa-Associated Bacterial Microbiome of the Gastrointestinal Tract of Weaned Pigs and Dynamics Linked to Dietary Calcium-Phosphorus
Source: PLoS One. 2014 Jan 23;9(1):e86950. doi: 10.1371/journal.pone.0086950 (PMC3900689; doi:10.1371/journal.pone.0086950)
Supplement: Table S6 — Relative abundances of the 30 most abundant bacterial genera in the gastrointestinal tract of pigs fed wheat-barley or corn diets including adequate or high Ca-P. (PDF) [file pone.0086950.s010.pdf]

Table S6. Relative abundances of the 30 most abundant bacterial genera in the gastrointestinal tract of pigs fed wheat-barley or corn diets including adequate or high Ca-P. Values are least squares and standard error of the mean (SEM). Statistically significant shifts are highlighted in orange, trends in pale orange.

	Relative abundance [%]					*p*-Value		
	Wheat-Barley		Corn					
	Adequate Ca-P	High Ca-P	Adequate Ca-P	High Ca-P	SEM	Cereals	Ca-P	Cereals × Ca-P
**Stomach**								
Lactobacillus	51.6	66.7	37.9	60.7	8.47	0.256	0.035	0.658
Prevotella	17.4	12.3	22	11.3	4.20	0.668	0.073	0.515
Helicobacter	0.2	0.2	6.8	0.1	3.25	0.324	0.315	0.312
Bacteroides	2.2	0.8	2.4	3.1	1.21	0.303	0.806	0.392
Campylobacter	0.2	<0.1	0.5	0.1	0.17	0.379	0.090	0.613
Clostridium XI	1.4	0.3	0.8	1.4	0.44	0.554	0.568	0.072
Acinetobacter	3.6	1.6	7.7	0.3	3.77	0.710	0.225	0.477
Paraprevotella	1.8	0.9	1.4	1.4	0.71	0.966	0.564	0.564
Pseudomonas	1.3	0.8	2.4	1.1	0.65	0.287	0.155	0.468
Escherichia-Shigella	1.4	0.8	1.6	1.6	0.56	0.424	0.601	0.592
Streptococcus	0.6	0.6	0.7	0.9	0.33	0.610	0.722	0.844
Lachnospiracea incertae sedis	0.9	0.3	0.5	0.6	0.22	0.878	0.253	0.213
Clostridium sensu stricto	0.8	0.3	0.6	0.9	0.35	0.506	0.732	0.253
Faecalibacterium	0.8	0.4	0.7	0.7	0.21	0.511	0.345	0.341
Clostridium XIX	<0.1	<0.1	<0.1	<0.1	0.00	0.202	0.619	0.690
Haemophilus	0.6	0.3	0.7	1.0	0.29	0.231	0.964	0.252
Aminiphilus	0.7	0.3	0.4	0.7	0.25	0.808	0.878	0.196
Xylanibacter	0.6	0.2	0.5	0.4	0.20	0.989	0.244	0.430
Fusobacterium	0.5	0.3	0.4	0.7	0.24	0.381	0.778	0.316
Citrobacter	0.5	0.3	0.3	0.1	0.12	0.198	0.106	0.816
Coprobacillus	0.5	0.3	0.4	0.7	0.26	0.468	0.744	0.362
Dialister	0.6	0.4	1.0	1.0	0.39	0.180	0.879	0.811
Klebsiella	0.3	0.2	0.3	0.4	0.12	0.447	0.879	0.810
Proteus	0.3	0.2	0.4	0.6	0.21	0.286	0.899	0.410
Oscillibacter	0.5	0.2	0.4	0.4	0.11	0.704	0.280	0.123
Phocaeicola	0.5	0.2	0.5	0.2	0.15	0.898	0.042	0.874
Acidovorax	0.3	0.5	0.6	0.2	0.21	0.951	0.581	0.161
Asteroleplasma	0.3	0.1	0.1	0.1	0.10	0.486	0.288	0.391
Sediminitomix	0.3	0.1	0.3	0.2	0.13	0.795	0.287	0.496
Rikenella	0.4	0.1	0.2	0.3	0.13	0.560	0.428	0.136
**Ileum**								
Lactobacillus	12.7	12.4	12.1	16.9	3.07	0.530	0.471	0.428
Prevotella	14.6	8.4	10.1	7.7	4.50	0.570	0.346	0.687
Helicobacter	22.8	29.2	41.9	24.9	12.24	0.550	0.672	0.348
Bacteroides	4.1	4.9	6.8	3.8	2.64	0.766	0.682	0.462
Campylobacter	9.0	1.1	0.6	0.1	2.94	0.122	0.170	0.214
Clostridium XI	2.9	9.3	2.4	8.2	2.82	0.771	0.042	0.918
Acinetobacter	3.8	1.4	0.2	2.6	1.70	0.492	0.989	0.168
Paraprevotella	1.0	1.5	1.0	1.1	0.64	0.722	0.685	0.787
Pseudomonas	3.8	3.4	1.9	2.9	0.92	0.200	0.724	0.464
Escherichia-Shigella	2.8	3.1	2.0	3.9	1.05	0.938	0.301	0.458
Streptococcus	0.8	<0.1	7.5	8.1	4.38	0.105	0.981	0.873
Lachnospiracea incertae	0.6	0.6	0.3	0.4	0.17	0.232	0.831	0.620

sedis											
Clostridium sensu stricto	1.2	1.7		0.8	1.9		0.38		0.773	0.059	0.462
Faecalibacterium	0.6	0.7		2.4	0.8		1.01		0.369	0.463	0.431
Clostridium XIX	3.6	<0.1		0.1	<0.1		1.69		0.365	0.327	0.341
Haemophilus	0.9	1.4		0.9	1.3		0.38		0.787	0.249	0.855
Aminiphilus	0.7	0.9		0.4	0.7		0.24		0.324	0.287	0.799
Xylanibacter	0.3	0.3		0.7	0.5		0.21		0.195	0.472	0.754
Fusobacterium	0.9	1.2		0.5	0.7		0.34		0.276	0.466	0.887
Citrobacter	0.7	2.2		0.1	1.7		0.62		0.380	0.016	0.918
Coprobacillus	0.8	0.9		0.4	0.8		0.29		0.470	0.347	0.612
Dialister	0.1	0.6		0.2	0.2		0.26		0.534	0.338	0.321
Klebsiella	0.5	0.9		0.4	2.0		0.52		0.359	0.061	0.256
Proteus	0.6	0.9		0.4	0.5		0.24		0.252	0.483	0.782
Oscillibacter	0.5	0.4		0.2	0.4		0.15		0.292	0.808	0.349
Phocaeicola	0.5	0.2		0.2	0.1		0.19		0.343	0.411	0.467
Acidovorax	0.7	1.1		0.4	0.2		0.38		0.150	0.891	0.488
Asteroleplasma	0.5	0.3		<0.1	0.2		0.20		0.148	0.958	0.403
Sediminitomix	0.1	0.2		0.1	0.2		0.07		0.796	0.231	0.443
Rikenella	0.1	0.5		0.2	0.4		0.27		0.904	0.237	0.671
**Colon**											
Lactobacillus	9.8	12.1		7.9	8.8		2.85		0.368	0.583	0.804
Prevotella	38.4	42.4		40.4	44.8		5.56		0.692	0.454	0.976
Helicobacter	22.5	12.4		18	13.9		6.54		0.819	0.288	0.650
Bacteroides	0.1	0.2		0.1	0		0.09		0.167	0.842	0.523
Campylobacter	8.6	3.9		10.8	3.9		3.14		0.741	0.078	0.731
Clostridium XI	0.7	0.3		0.2	0.6		0.32		0.804	0.950	0.265
	<0.1	<0.1		<0.1	<0.		0.02		0.340	0.637	0.908
Paraprevotella	2.5	5.4		5.3	7.1		2.04		0.280	0.264	0.809
Pseudomonas	<0.1	<0.1		<0.1	<0.1		0.01		0.718	0.829	0.960
Escherichia-Shigella	0.1	0.1		0.1	0.1		0.03		0.413	0.395	0.777
Streptococcus	<0.1	<0.1		0.1	0.1		0.05		0.048	0.826	0.932
Lachnospiracea incertae sedis	0.6	0.8		0.4	0.9		0.21		0.929	0.087	0.584
Clostridium sensu stricto	0.1	0.2		0.1	0.1		0.06		0.243	0.552	0.982
Faecalibacterium	0.3	0.5		0.7	0.7		0.21		0.206	0.777	0.522
Clostridium XIX	<0.1	<0.1		<0.1	<0.1		0.06		0.272	0.272	0.223
Haemophilus	<0.1	<0.1		<0.1	<0.1		0.01		0.112	0.588	0.692
Aminiphilus	0.3	0.5		0.6	0.7		0.17		0.227	0.398	0.605
Xylanibacter	1.7	1.1		1.4	1.7		0.45		0.755	0.736	0.330
Fusobacterium	<0.1	<0.1		<0.1	<0.1		0.01		0.312	0.475	0.475
Citrobacter	<0.1	<0.1		<0.1	0.1		0.02		0.922	0.228	0.488
Coprobacillus	<0.1	<0.1		<0.1	<0.1		0.01		0.408	0.809	0.554
Dialister	0.8	0.4		0.2	0.3		0.22		0.134	0.596	0.252
Klebsiella	<0.1	<0.1		<0.1	<0.1		0.01		0.092	0.742	0.653
Proteus	<0.1	<0.1		<0.1	<0.1		0.01		0.224	0.509	0.991
Oscillibacter	0.5	0.5		0.4	0.5		0.10		0.608	0.860	0.533
Phocaeicola	0.6	1.3		0.9	1.1		0.39		0.920	0.253	0.567
Acidovorax	<0.1	<0.1		<0.1	<0.1		0.01		0.224	0.196	0.973
Asteroleplasma	0.9	0.4		0.7	1.7		0.46		0.257	0.583	0.147
Sediminitomix	0.7	1.4		0.5	1.0		0.41		0.426	0.155	0.824
Rikenella	0.6	1.3		1.2	1.0		0.50		0.741	0.606	0.390
