# Supplementary material for: Mucosa-Associated Bacterial Microbiome of the Gastrointestinal Tract of Weaned Pigs and Dynamics Linked to Dietary Calcium-Phosphorus
Source: PLoS One. 2014 Jan 23;9(1):e86950. doi: 10.1371/journal.pone.0086950 (PMC3900689; doi:10.1371/journal.pone.0086950)
Supplement: Text S1 — Supplementary information about sequence processing and SAS procedures. (PDF) [file pone.0086950.s011.pdf]

Text S1

Sequence processing: Triming

Primers, barcode sequences and sequences of low quality and length were trimmed with a minimum average quality score of 35 (using a window size of 50 bp), a minimum length of reads of 162 bp and a maximum allowed number of differences to primer sequence of two. Ambiguous bases were not allowed, the maximal homopolymer length was eight and the maximum allowed number of differences to the barcode sequence was one.

SAS procedures

The assigned OTUs and the diversity indices were subjected to ANOVA using the PROC MIXED of SAS (Statistical Analysis System 9.2, SAS Inst. Inc., Cary, NC, USA). To evaluate the effect of GIT sites, the SAS model included the options “fixed effects of intestinal site and experimental run” and “random effect of pig (n = 31) nested within a sow prior weaning (n = 16)”. The model considers every pig as experimental unit. The covariance structure of every experimental unit was modeled separately according to the smallest values of the fit statistics based on the Bayesian information criteria (BIC). To identify diet effects per GIT site, fixed effects in the model included “effects of Ca-P content, basal diet and their two-way-interaction” and “random effect of pig (n = 31) nested within a sow prior meaning (n = 16)”. This model also considers every pig as experimental unit. For the determination of any influenctial observation on the models, the Cook’s distance (Cook’s D) test was used to check for outliers. Because no outliers could be detected, the Cook’s distance test was removed from the final model. Means were reported as least-squares means ± standard error of the mean (SEM). If an interaction between basal diet and Ca-P content occurred, multiple comparisons of least-squares means were performed using “pdiff option” of SAS. Degrees of freedom were approximated using Kenward-Rogers method (ddfm = kr).
